# Sensory Encoding Alternates With Hippocampal Ripples across Cycles of Forebrain Spiking Cascades

**DOI:** 10.1002/advs.202406224

**Published:** 2025-02-27

**Authors:** Yifan Yang, David A. Leopold, Jeff H. Duyn, Grayson O. Sipe, Xiao Liu

**Affiliations:** ^1^ Department of Biomedical Engineering The Pennsylvania State University University Park PA 16802 USA; ^2^ Neurophysiology Imaging Facility National Institute of Mental Health National Institute of Neurological. Disorders and Stroke and National Eye Institute National Institutes of Health Bethesda MD 20892 USA; ^3^ Section on Cognitive Neurophysiology and Imaging Systems Neurodevelopment Laboratory National Institute of Mental Health National Institutes of Health Bethesda MD 20892 USA; ^4^ Advanced MRI Section Laboratory of Functional and Molecular Imaging National Institute of Neurological Disorders and Stroke National Institutes of Health Bethesda MD 20892 USA; ^5^ Department of Biology The Pennsylvania State University University Park PA 16802 USA; ^6^ Institute for Computational and Data Sciences The Pennsylvania State University University Park PA 16802 USA

**Keywords:** brain states, global signal, hippocampal ripples, low‐frequency neuronal population dynamics, neural coding

## Abstract

The brain's response to external events depends on its internal arousal states, which are dynamically governed by neuromodulatory systems and have recently been linked to coordinated spike timing cascades in widespread brain networks. At rest, both arousal fluctuations and spiking cascades are evident throughout the forebrain and play out over multisecond time scales. Here, by analyzing large‐scale neural recording data collected by the Allen Institute, it is demonstrated that these intrinsic processes persist across the mouse brain even during periods of continuous visual stimulation. In the stationary animal, each quasi‐periodic cascade cycle systematically influenced 1) the efficacy of encoding in visually responsive brain areas and 2) the incidence of memory‐related hippocampal ripples. During this cycle, the phase of high arousal is marked by high efficiency in visual encoding whereas the phase of low arousal is marked by the occurrence of hippocampal ripples. However, during bouts of active locomotion, this cycle is abolished and brain maintained a state of elevated visual coding efficiency, with ripples being nearly absent. It is hypothesized that the infra‐slow cascade dynamics reflect an adaptive cycle of alternating exteroceptive sensory sampling and internal mnemonic function that persistently pervades the forebrain, only stopping during active exploration of the environment.

## Graphical abstract

This study reveals widespread, coordinated brain activity during passive visual stimulation in mice. Sensory coding, phase‐locked with the periodic dynamics, shows an inverse relationship to hippocampal ripples. The results suggest that periodic brain activity spurs alteration between two distinct operational modes of brain, facilitating exteroceptive sensory sampling and internal mnemonic processes, respectively.

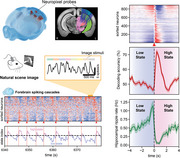

## 1 Introduction

A core function of the brain is its ability to encode and process external stimuli in both primary and higher‐level sensory areas. However, much evidence suggests that the magnitude and reliability of sensory responses in the cerebral cortex and elsewhere depend on the brain's state of arousal.^[^ ^1^, ^2^, ^3^, ^4^ ^]^ This dependency is most dramatically evident under different states of consciousness, such as waking, sleep, and anesthesia, when the same sensory stimulus can elicit very different neural responses.^[^ ^2^, ^3^ ^]^ Even during wakefulness, sensory responses can be highly variable and dependent on momentary arousal level that could be critically determined by ongoing brain activity (see Table S1, Supporting Information for a summary of related studies).

One particularly salient example of arousal state fluctuations affecting sensory responses during wakefulness comes from electrophysiological studies in mice. Cortical responses to visual and somatosensory stimuli are greatly enhanced when mice actively engage in behaviors such as whisking or locomotion, compared to when they are quiescently awake.^[^ ^5^, ^6^, ^7^, ^8^, ^9^ ^]^ Such sensory enhancements generally occur during aroused brain states characterized by desynchronized cortical activity.^[^ ^10^, ^11^, ^12^, ^13^, ^14^ ^]^ In addition, startling an animal with an air puff transiently desynchronizes cortical activity and increases visual responses in the absence of locomotion.^[^ ^15^ ^]^ Similar sensory response effects have been elicited by manipulating the noradrenergic and cholinergic systems, which are thought to underlie changes in arousal.^[^ ^16^, ^17^, ^18^, ^19^, ^20^ ^]^ Thus, the arousal state impacts how the brain processes external stimuli during wakefulness. Interestingly, in the absence of overt active behaviors and external arousal modulators, a large variability of sensory responses was observed across trials lasting several seconds.^[^ ^21^, ^22^ ^]^ This variability has been linked to pre‐stimulus ongoing brain activity^[^ ^21^, ^22^ ^]^ and spontaneous arousal changes.^[^ ^23^, ^24^, ^25^ ^]^ Nevertheless, the nature and source of the spontaneous multi‐second fluctuations in ongoing brain activity and arousal, such as whether they occur purely randomly or are organized in a highly structured manner to coordinate with other non‐sensory brain processes, remain unclear.

In the absence of external stimuli, the brain exhibits widespread and pronounced spontaneous activity that transpires over multiple seconds. These coordinated bouts of neural activity have been observed in human fMRI and monkey electrocorticography (ECoG).^[^ ^26^, ^27^ ^]^ Detailed analyses have shown that these neural events usually take the form of relatively slow, spatiotemporal waves that propagate across the cortical hierarchy over periods spanning multiple seconds.^[^ ^27^, ^28^, ^29^ ^]^ Similar events have also been observed in large‐scale single‐unit recordings in the mouse,^[^ ^30^, ^31^ ^]^ where temporally coordinated spiking events are evident in cortical and subcortical structures. In fact, in our previous work,^[^ ^31^ ^]^ we found that up to 70% of spike‐sorted units from various cortical and subcortical areas are entrained into highly‐structured temporal sequences, or cascades, that recur quasi‐periodically. With each occurrence, individual neurons show spike rate modulation at a consistent phase of the cascade. These observations of large‐scale brain activity using different methods appear tightly linked to moment‐to‐moment changes in central and autonomic arousal levels. For instance, global fMRI peaks and waves are accompanied by deactivation of subcortical arousal‐regulating centers, along with a simultaneous increase in delta‐band (1–4 Hz) power observed in both monkey intracranial EEG and human scalp EEG recordings.^[^ ^26^, ^27^ ^]^ Additionally, spiking cascades are phase coupled to arousal changes measured by pupil size and delta‐band (1‐4 Hz) cortical brain activity.^[^ ^31^ ^]^

While these highly structured, brain‐wide events are evident during wakeful rest, it is not known whether they also occur during passive sensory stimulus processing and/or active behavior. If they do, such events might affect the efficacy of stimulus encoding or relate to the known modulation of encoding by locomotion. Importantly, their influence on sensory encoding may intertwine with their observed dynamic connection to memory‐related neural events.^[^ ^31^ ^]^ Resolving these possibilities will improve our understanding of how the brain balances the distinct tasks of external stimulus processing and internally regulated functions like memory, which have competing demands on neural resources.

To address these questions, we examined spiking activity collected from neuronal populations in 44 cortical and subcortical areas in mice during quiescent rest, visual stimulation, and active locomotion, using the Neuropixels dataset from the Allen Institute.^[^ ^32^ ^]^ We found that during periods of visual stimulation in the absence of locomotion, spiking cascades persisted and remained similar to those observed during quiescent rest. Visual responses were systematically modulated across each cascade cycle. The accuracy of visual coding was greatly enhanced in a ∼2 s phase associated with high arousal whereas suppressed in the remaining low arousal phase. Hippocampal sharp‐wave‐ripple complexes (SPW‐Rs), which are critical for memory function,^[^ ^33^, ^34^, ^35^ ^]^ were oppositely modulated across the high‐ and low‐arousal phases over the cascade cycle. Strikingly, active locomotion abolished cascade sequences and maintained neural activity in the high‐arousal phase. Together, these results suggest that highly structured cascades of spiking are entrained by a quasi‐periodic, arousal‐related cycle that affects activity across the forebrain. We hypothesize that this cycle spurs the alternation between two distinct operational modes of the brain, subserving sensory and memory functions, respectively.

## 2 Results

**Figure 1:**
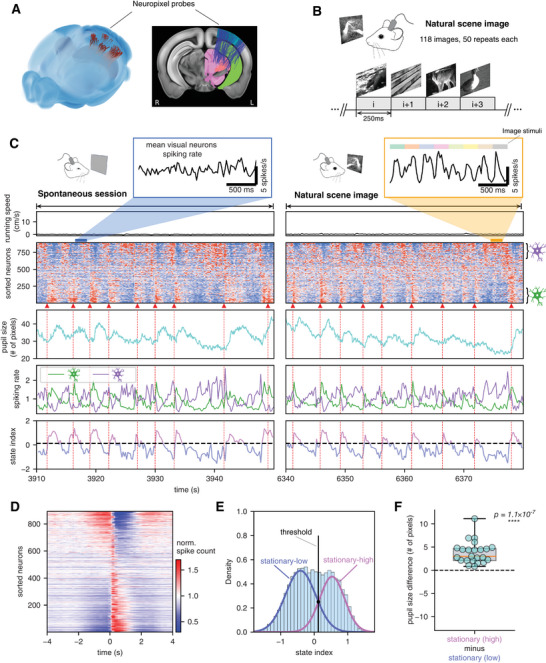
Spiking cascades of arousal relevance persist during continuous visual stimulation. A) Locations of 183 neuropixel probes from 32 mice in the Allen Mouse Brain Common Coordinate Framework (version 3). Left: the 3D representation of probe insertion in mouse brain. Right: the 2D projection of the probes onto a middle brain slice highlights the major recording sites: visual cortex (blue), hippocampus (green), and thalamus (pink). B) Illustration of natural scene image session where grayscale images were continuously presented to mice for 250 ms each in random order through a monitor. The 118 images were each presented 50 times, across 3 different sessions in every mouse. C) Example data from a representative mouse under two different conditions. Left column: A 40 s stationary period of spontaneous session without stimulation. In the absence of running (first row), the neuronal spikes were organized in the form of spiking cascades (second row: neurons were sorted by the principal delay profile derived by a data‐driven method) with sequential activation from the negative‐delay neurons (fourth row: purple colored) to the positive‐delay neurons (fourth row: green colored). The spiking cascade is phase‐coupled to changes in pupil diameter (third row). A state index was then defined based on the relative activation level of positive‐delay neurons and negative‐delay neurons with pink/blue colors denoting the high/low arousal states respectively (fifth row). Right column: A 40 s stationary period of the nature image stimulation session that showed similar spiking cascades during visual stimulation. The pop‐up panels at the top show the mean spiking rate of all visual area neurons in example 2 s time segments, which was phase‐locked to stimuli in the image stimulation session (orange) but did not show any systematic pattern in the spontaneous session without stimulation (blue). Although a large proportion of neurons are located within visual areas (n = 339, 38%) and are responsive to image stimuli (n = 290, 32%), this does not alter the cascade pattern observed during visual stimulation. See Figure S6 (Supporting Information) for more details. D) The average pattern of the spiking cascade in the representative mouse. E) Distribution of state index in stationary periods (light‐blue‐colored) from a representative mouse. The stationary distribution is fitted with a two‐classes Gaussian mixture model, yielding the fitted curves for the stationary‐high state (pink‐colored) and stationary‐low state (blue‐colored), as well as the optimal boundary between the two (black dash line). F) The boxplot of pupil diameter difference between the stationary‐high and stationary‐low states for mice with pupil data. Each dot represents a mouse and the one‐sample *t*‐test (two‐sided, *N* = 23) is used to test significance.

To investigate the relationship of intrinsic spiking cascades with visual coding, arousal, and hippocampo‐cortical dynamics, we used the Visual Coding – Neuropixels dataset from the Allen Institute.^[^ ^32^, ^36^ ^]^ We analyzed the spiking activity of ∼22 000 spike‐sorted units (hereafter referred to as neurons) recorded from 32 mice (683 ± 140 neurons per mouse, mean ± SD, see Table S2, Supporting Information and Methods for details), spread across 44 brain regions, mostly in the visual cortex, hippocampus, and thalamus (**Figure** **1**A). We focused on 7 types of experimental sessions, including 3 involving natural scene visual stimuli, 3 involving drifting‐gratings, and one spontaneous session without stimulation (Figure S1A, Supporting Information).

### 2.1 Spiking Cascades Pervade the Cortex during Visual Stimulation

We first arranged the simultaneously recorded neurons according to their principal delay profile, which reflects the dominant direction of spontaneous brain‐wide infra‐slow sequential activations, known as spiking cascades.^[^ ^31^ ^]^ Briefly, we segmented spiking data according to the troughs of the global mean signal, defined a delay profile for each segment to describe relatively delays of neurons’ peak activations, and then applied singular value decomposition (SVD) to extract the first principal component of all delay profiles, termed as the principal delay profile (see Methods for details). We compared periods with and without visual stimulation, both in the absence of locomotion (“stationary behavior”) (Table S3, Supporting Information). Against expectation, the cascades of spiking activity pervaded brain activity in a manner that was both qualitatively and quantitatively similar between the two conditions (Figure 1C and Figure S1 (Supporting Information); see also Figures S2 and S3 (Supporting Information) for additional examples under stimulation). Within each cascade, individual neurons had a fixed temporal relationship to one another, with the average over all cascades in a representative session shown in Figure 1D. The similarity of cascade dynamics with and without visual stimulation is reflected in highly similar (*r* = 0.836 ± 0.08, mean ± SD; *p* = 8.5 × 10^−84^; Figure S1D, Supporting Information) principal delay profiles across the two conditions.

We defined a state index, quantified by the difference in activation levels between the positive‐delay (green symbols in Figure 1C) and negative‐delay neurons (purple symbols in Figure 1C). These neuronal subpopulations reside at the two extremes of the principal delay profile and are activated during distinct phases of each cascade cycle (Figure 1C,D; Figure S4, Supporting Information). The two subpopulations exhibit regional preferences similar to what has been observed during rest:^[^ ^31^ ^]^ positive‐delay neurons are predominantly found in the thalamus whereas negative‐delay neurons are primarily located in the hippocampus, particularly CA1 region. Cortical regions have a balanced distribution of both types, but most cortical neurons belong to neither group (Figure S5, Supporting Information). The state index served as a proxy for the internal state of the brain entrained to each cascade. It showed a bimodal distribution during the stimulated stationary periods, suggesting the existence of two distinct brain states (Figure 1E). Based upon the reliable variation of the state index with pupil size changes (Figure 1F), we refer to the two states as low‐arousal and high‐arousal, which correspond well to distinct phases of the cascades (bottom row in Figure 1C). During high arousal state, the pupil was dilated relatively and positive‐delay neurons showed their highest activity, whereas during low arousal state the pupil was constricted and negative‐delay neurons showed their highest activity.

These findings demonstrate that the spiking cascade dynamics observed previously during quiescent, unstimulated rest persist in approximately the same form during periods of continuous and intense visual stimulation when the animal is stationary. These apparently self‐generated dynamics, which are not time‐locked to much more rapid (i.e., 250 ms) external stimulus presentations, cause the brain to engage in quasi‐periodic fluctuations with intervals of 3–10 s (cascade duration: 5.6 ± 1.6 s, mean ± standard deviation, Figure S1E, Supporting Information). Within each cycle, the brain alternates between two distinct states that vary in their arousal levels.

### 2.2 Stimulus Encoding and Hippocampal Ripples are Inversely Modulated during each Cascade Cycle

**Figure 2:**
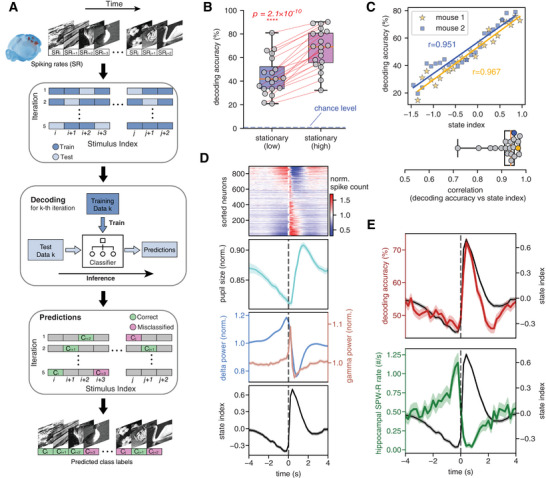
Population encoding of visual stimuli and hippocampal ripple rate tightly follows the cascade dynamics in inverse ways. A) Pipeline for training a machine learning model to predict visual stimuli based on population spike data. The 118 × 50 stimulation trials (250 ms, 50 repetitions for each of 118 image stimuli) were shuffled and evenly split into 5 groups with each covering 10 repetitions of all the 118 stimuli. For each iteration, an SVM‐based decoder was trained with data of 4 groups and then used to make inferences on the remaining group. Every trial was evaluated and labeled either correctly classified or misclassified after 5 iterations. B) Box plot showing the decoding accuracy summarized for different conditions. Each dot represents a mouse and the two‐sided pairwise *t*‐test was used for the significance test (*N* = 20 mice). The blue dashed line marked the chance level of 0.85%. C) The linear relationship between state index and decoding accuracy is shown for two example mice (Top) and also summarized in a box plot for all mice (Bottom), where the yellow and blue dots represent the example mice. The linear relationship was derived from the data during the stationary periods of continuous visual stimulation. D) Various behavior and neuronal signals averaged over the 8‐s cascade cycle, from top to bottom showing the averaged cascade pattern, pupil size, LFP band powers, and the state index. Note the averaged cascade pattern is from the representative mouse. E) The opposite modulations of the decoding accuracy (top) and hippocampal sharp wave ripples (SPW‐Rs) rate (bottom) across the cascade cycle during stationary periods with natural image stimulation. All time series data are shown as mean ± SEM from all cascade events (*N* = 1401) across all 32 mice.

We investigated how the cascade dynamics may affect the encoding of visual stimuli among recorded neuronal populations. To study this, we employed machine learning models to measure the precision of visual information encoding and its dependency on neural population dynamics. We trained support vector machine (SVM) decoders to predict the image identity based on the neuronal spiking activity it evoked. The decoding results were assessed with 5‐fold cross‐validation and the correctness of the predictions was documented for each of the 118 stimuli × 50 repeated presentations in its test phase (**Figure** **2**A, also see Methods for details). The prediction accuracy of the decoders serves as a measure of encoding performance across different conditions and reflects the efficacy of visual sensory processing.

The encoding of visual stimuli was strongly affected by the internal state defined by population spiking dynamics. On average, the decoding accuracy showed a striking difference (23.0 ± 8.5%, *p* = 2.1 × 10^−10^, *N* = 20 mice, paired *t*‐test) between the two stationary arousal states defined through population spiking activity (Figure 2B). Given that the two states were defined based on the state index, we directly examined the relationship between the state index and decoding accuracy. This analysis revealed a strong and robust linear association (*r* = 0.975, *p* = 3 × 10^−21^; all mice data; Figure S7A, Supporting Information) between the two, which was highly reproducible across individual mice (Figure 2C). In contrast, the decoding accuracy exhibits an inverse‐U relationship with pupil diameter (Figure S7B, Supporting Information), consistent with a previous study.^[^ ^24^ ^]^ The discrepant dependency of decoding accuracy on pupil size and state index may be attributed to their non‐linear relationship (Figure S7C, Supporting Information), as well as relative time delays (Figure S4E, Supporting Information).

During stationary periods of visual stimulation, these different modes of stimulus encoding were attached to different phases of the cascade cycle. The encoding accuracy was temporally entrained, along with multiple arousal measures, to the occurrence of each cascade event (Figure 2D,E). Arousal measures such as pupil size, delta (<4 Hz) and gamma (55–65 Hz) local field potential (LFP) power, and the previously computed state index, were systematically modulated with each cascade cycle (Figure 2D). In particular, the decoding accuracy and state index were modulated in a highly similar way, with their fast descending phase (0.3‐2 seconds with respect to the cascade center) coinciding actually with the pupil dilation. Importantly, simultaneous measurements from the hippocampus indicated that sharp wave ripples (SPW‐Rs) were modulated in an antagonistic way to both: the ripple rate was the highest at the troughs of state index and decoding accuracy whereas it decreased to almost zero at state index peaks (Figure 2E).

**Figure 3:**
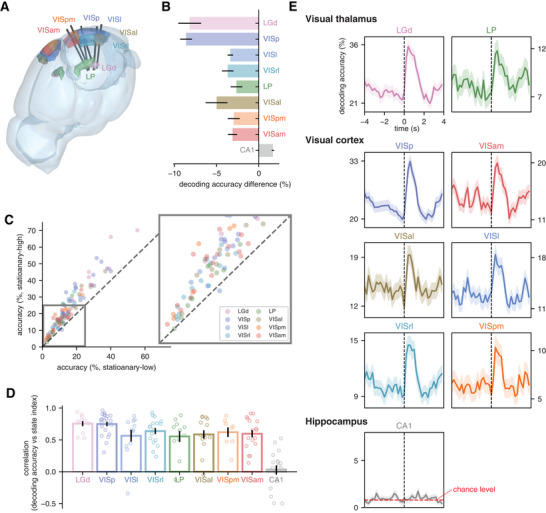
Cascade phase dependent visual coding is evident in all visual areas. A) Schematic layout of Neuropixel probes relative to their target visual regions, including primary visual cortex (VISp) and five high‐order visual cortical areas, i.e., latero‐medial area (VISl), antero‐lateral area (VISal), rostro‐lateral area (VISrl), postero‐medial area (VISpm) and antero‐medial area (VISam) (image credit: the Allen Institute). The probes with insertion depth up to 3.5 mm into the brain also record the spiking activity within two visual thalamic nuclei, i.e., the lateral posterior nucleus (LP) and the lateral geniculate nucleus (LGN), as well as other regions traversed by the probes, e.g., the hippocampus. B) A bar plot showing the change in decoding accuracy resulting from excluding a specific region. C) A comparison of the accuracy of region‐specific decoders, which were trained with data of a specific brain region, between the stationary‐high and stationary‐low states. Each colored dot represents a specific visual region from a single mouse. D) A box plot showing the linear relationship between the state index and the accuracy of region‐specific decoders, similar to Figure 2C. Each dot represents a mouse with the corresponding region recorded. E) The region‐specific decoding accuracy over the 8‐s spiking cascade cycle from all the mice with the corresponding region recorded (*N* = 1401). The red dashed line marks a chance level equal to 0.85%.

We then investigated the effect on visual encoding in multiple different visual regions of the mouse brain, while using the hippocampus as a control region. Performance of the decoder was able to draw upon all visual areas, with the lateral geniculate nucleus (LGN) and primary visual cortex (VISp) showing the strongest contribution, such that their exclusion led to the strongest reduction in decoding accuracy (**Figure** **3**B). Analysis of the weight contribution in the SVM models (Figure S8A, Supporting Information) and the accuracy of decoders trained with single‐region data (Figure S8B, Supporting Information) yielded similar results.

Importantly, the accuracy of each single‐region decoder was consistently higher during the stationary‐high state than the stationary‐low state (Figure 3C) and actually correlated with the state index (Figure 3D), indicating that neurons in each of the areas were affected by the cascade fluctuations in qualitatively similar ways. This is also evident in the dynamics of each area's encoding accuracy time course over the cascade cycle (Figure 3E). These results were consistent across different choices of decoder models, i.e., logistic regression (Figure S9, Supporting Information) and multilayer perceptron (Figure S10, Supporting Information), and remained robust when using other time bin sizes (i.e., 50 ms (Figure S11, Supporting Information), 100 ms (Figure S12, Supporting Information)) for quantifying neural codes. In each case, shuffling the stimulus labels completely abolished decoder performance (Figure S13, Supporting Information).

### 2.3 Locomotion Abolishes Cascade Dynamics during Visual Presentation

**Figure 4:**
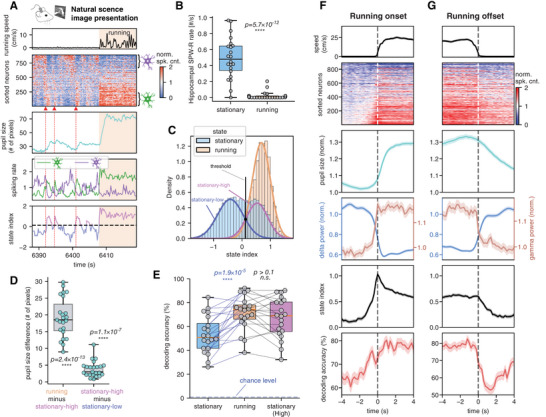
Running sustains visual coding efficiency and abolishes hippocampal sharp‐wave ripples. A) A 30 s period of the natural image session at the transition to a running episode. The transition is accompanied by the cessation of spiking cascades, a large increase in pupil diameter, sustained activation/deactivation of positive‐delay/negative‐delay neurons, and a high state index. B) The occurrence rate of the hippocampal SPW‐R summarized for stationary (light‐blue) and running (orange) respectively, which are significantly different from each other (two‐sample *t*‐test, two‐sided). C) The state index distribution of the representative mouse during running periods (orange) as compared with the stationary periods (light blue). D) The boxplot of pupil diameter differences for 23 mice with pupil data. The averaged pupil diameter differences were separately summarized between the running and the stationary‐high state periods, and between the stationary‐high and stationary‐low states. Each dot represents a mouse and the one‐sample *t*‐test (two‐sided, *N* = 23 mice) is used for the significance test. E) The decoding accuracy summarized for the running periods and the two stationary states. Each dot represents a mouse and the pairwise *t*‐test was used for the significance test (two‐sided, *N* = 20). F) Changes of behavior and neuronal signals and visual coding accuracy at the running onset. The data were averaged over all onset events (*N* = 465) during natural scene image sessions across all 32 mice, from top to bottom showing the running speed, averaged cascade pattern, pupil size, delta/gamma power, state index, and neural decoding accuracy. G) The change of signals at the running offset (*N* = 401), the same as in (F).

Given previous findings that locomotion affects visual information processing,^[^ ^5^, ^9^, ^15^, ^19^ ^]^ we investigated whether the cascade dynamics and visual coding accuracy would be affected by the mouse's running behavior. We found that, during running episodes, spiking cascades nearly vanished and the cyclical nature of arousal seen in the absence of locomotion ceded to a continuous condition of high‐arousal, with negative‐delay neurons continually suppressed whereas the positive delay neurons showing sustained activation (**Figure** **4**A). Hippocampal ripples were nearly abolished (Figure 4B). In contrast to the stationary periods of visual stimulation, the computed state index during active running and visual stimulation had a single peak in its distribution (light yellow, Figure 4C), corresponding to the stationary‐high arousal state. Accordingly, visual coding accuracy during running was much higher than the stationary periods overall (Figure 4E and *p* = 1.9 × 10^−5^, paired *t*‐test, *N* = 23 mice) but similar to the stationary‐high state (*p =* 0.13, paired *t*‐test, *N* = 23 mice). Nevertheless, the running periods were associated with much larger pupil size than the stationary‐high periods (Figure 4D and *p* = 2.4 × 10^−13^, paired *t*‐test, *N* = 23 mice).

Given this clamping to a high‐arousal state during periods of locomotion, the onset and offset of running provided an additional perspective on the temporal dynamics of the associated brain state changes. We found that decoding accuracy and state index preceded running onset by ≈1.5 s and took ≈1.5 s to slowly decrease back to the baseline after the cessation of running (Figure 4F,G), suggesting a tight association between the sensory coding and neural dynamics but their weaker association with locomotion. The pupil dilation/constriction showed similar, albeit sluggish, changes at both running onsets and offsets.

### 2.4 Cascades Affect Visual Coding by Modulating the Magnitude of Sensory Responses

**Figure 5:**
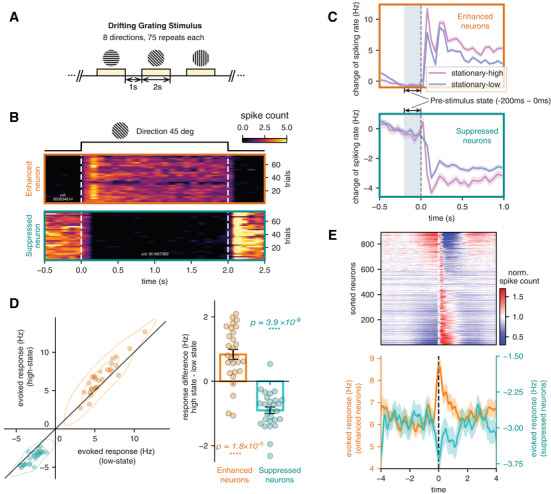
Spiking cascade modulates single‐neuron responses to change the signal‐to‐noise ratio. A) Illustration of stimulus presentation in the drifting‐grating session. Oriented drifting gratings were repeatedly presented to mice for two seconds following a one‐second blank period. B) Examples of two types of neurons (top: drifting grating enhanced neuron; bottom: drifting grating suppressed neuron) that showed opposite responses to a drifting grating stimulus. C) The mean responses of enhanced neurons (top) and suppressed neurons (bottom) in a representative mouse differ with respect to the pre‐stimulus state (−200 to 0 ms before stimulus onset) defined by the state index. The results are shown as mean ± SEM. D) Response amplitude compared between the trials with different pre‐stimulus states for both enhanced (orange) and suppressed (teal) neurons. Single neuron response is quantified as the mean spiking rate difference between stimulus onset (0 to 400 ms) and baseline (−800 to 0 ms). Each dot represents a mouse, the one‐sample *t*‐test is used for the significance test (two‐sided, *N* = 28). E) The mean responses of the enhanced (orange) and suppressed (teal) neurons are dependent on the phase of their pre‐stimulus period in the cascade cycle (bottom). The 160 time bins between −4 to 4 s have different numbers of trials ranging between 82 to 168. The averaged cascade pattern (top) is from the representative mice.

To understand the basis of changes in population encoding of visual information, we examined how single‐neuron responses are modulated by the cascade dynamics. For this purpose, we focused on the drifting‐grating stimulation sessions, which include a 1‐sec baseline period between stimulations and thus enable the estimation of evoked spiking response amplitudes (**Figure** **5**A). Confirming the findings above, we found that the orientation and temporal frequency of drifting‐grating stimuli can be more accurately decoded from the neuronal data of the stationary‐high state than that of the stationary‐low state (Figure S14, Supporting Information). Inspecting single‐neuron responses, we identified two groups of neurons showing significant but opposite responses to the drifting‐grating stimuli (enhanced and suppressed neurons in Figure 5B). Neurons may respond to more than one grating direction, but the type of response (enhanced or suppressed) was largely consistent (Figure S15A, Supporting Information).

We next investigated how the brain state affects single neuron evoked responses. Although the drifting‐grating responding neurons showed no systematic overlap with the negative‐ and positive‐delay neurons, i.e., the neurons exhibiting significant modulation in their activities across the cascade, we excluded the drifting‐grating responding neurons classified also as either negative‐ or positive‐delay neurons for subsequent analyses to avoid any potential confounding (Figure S15B, Supporting Information). We found that the pre‐stimulus state (−200−0 ms) significantly affected the amplitude of evoked responses. The stimuli presented right after the stationary‐high state elicited larger responses of the enhanced neurons but decreased spikes of the suppressed neurons to a larger extent, compared with those presented right after the stationary‐low state. This is evident in trial averages (Figure 5C) and across individual mice (Figure 5D). A similar result was also observed between the running and stationary periods (Figure S15C,D, Supporting Information). Importantly, after sorting the trials according to the location of their pre‐stimulus period in the cascade cycle, the response of the drifting grating enhanced and suppressed neurons clearly showed opposite modulations across the cascade cycle in a pattern similar to what we observed with population decoding accuracy (Figure 5E). These changes in single‐neuron responses could potentially affect the signal‐to‐noise (SNR) ratio of visually evoked responses, and thus influence the efficacy of visual coding over the cascade cycle.

## 3 Discussion

Here we show that the mouse brain, during stationary periods of passive visual stimulation, exhibited quasi‐periodic state fluctuations indicated by stereotyped population dynamics spanning several seconds. These dynamics, previously observed exclusively during rest, manifest as cyclical spiking cascades entraining the activity of a high proportion of neurons in the cortex, hippocampus, and thalamus. These cascades are accompanied by changes in hippocampal ripple activity and arousal indicators, such as pupil size. Interestingly, the cascade strongly influences the brain's capacity to accurately encode visual information, in a manner opposite to its effect on the hippocampal ripples. Moreover, locomotion abolished both the cascade cycles and hippocampal ripples, leading to prolonged periods of high arousal and efficient visual encoding. The results thus cast new light on the nature of neural variability in behaving animals.

We show that ongoing dynamics responsible for the moment‐to‐moment variability in neural responses are intricately organized and involve neural populations distributed across the brain. It is generally appreciated that an identical stimulus can lead to different neuronal responses depending on the brain state.^[^ ^1^, ^5^, ^11^, ^15^, ^37^ ^]^ This variability can play out over multiple time scales, including over a period of several seconds.^[^ ^15^, ^21^, ^25^ ^]^ Previous efforts to link this response variability to ongoing pre‐stimulus activity^[^ ^21^, ^22^ ^]^ have not focused on how the brain‐wide neural dynamics might contribute to the observed variability in local responses.^[^ ^38^, ^39^ ^]^ While arousal fluctuations are recognized as likely contributing to variable sensory responses,^[^ ^23^, ^31^ ^]^ the precise underlying network mechanisms are still poorly understood. The proposed state index, as a 1D representation of brain‐wide, highly organized cascade dynamics, may also serve as a low‐dimensional latent variable hypothesized to modulate the extent of time‐warped spike timing patterns on the millisecond scale.^[^ ^40^ ^]^

The finding of cyclical cascades pervading the brain during rest^[^ ^31^ ^]^ suggests that arousal fluctuations are fundamentally related to the phase of these cascades. Thus, in contrast to previous studies seeking to link sensory response variability to arousal fluctuations measured by pupil diameter changes,^[^ ^23^, ^24^, ^25^ ^]^ we focused on the role of cyclical brain‐wide cascades on population visual encoding. Our findings of persisting cascades during periods of visual stimulation, and their striking relationship with sensory encoding, indicate a fundamental signature and perhaps mechanism governing brain states. These findings have implications for understanding the neural basis of similar large‐scale brain dynamics measured using other methods such as fMRI and ECoG, which have been described as infra‐slow waves propagating along a cortical hierarchical gradient.^[^ ^27^, ^28^, ^29^, ^41^ ^]^ Like the cyclical spiking cascades measured in the present study, these propagating waves are also coupled to arousal fluctuation on the timescale of seconds, and could be the macro‐scale representation of cascade dynamics.

The influence of endogenous cascade dynamics over sensory encoding may have important functional implications. For example, alternating between modes of efficient stimulus encoding and hippocampal ripple activity may be relevant for mechanisms of learning and memory. Previous work has proposed that the alternation of distinct brain states serves memory consolidation across sleep,^[^ ^42^ ^]^ with cholinergic neuromodulation being a central component. According to this view, elevated cholinergic tone characteristic of wakefulness, prioritizes the encoding of new information into the hippocampus, whereas a reduced tone during sleep favors memory consolidation from the hippocampus to the cortex.^[^ ^42^ ^]^ Increased cholinergic and adrenergic modulatory activity is indeed correlated with spontaneous pupil dilations^[^ ^25^, ^43^ ^]^ and enhanced excitability of visual neurons,^[^ ^17^, ^43^, ^44^, ^45^, ^46^ ^]^ which are similar to what we observed at the high‐arousal phase of the cascade. The endogenous cascades we describe here may therefore represent the same alternation between sensory encoding and mnemonic function but on shorter timescales and entirely during wakefulness. The intrinsic cascades could thus drive the seconds‐scale oscillation between two modes during wakefulness even under continuous sensory stimulation. Such quick and repeated switching between the two functional modes, which resembles the alternating of information forwarding and error back‐propagating during the learning of artificial neuronal networks,^[^ ^47^ ^]^ might be generally important for learning and memory.

Another conjecture on the cascade function is related to its sequential nature, in which neurons activated at similar phases are more synchronized. Individual cascades might serve as a reference event to help bind information presented closely in time. The new finding of the correspondence between the cascade sequence and the hippocampal replay sequence supports this hypothesis.^[^ ^48^ ^]^ Additional experiments are needed to test this exciting possibility.

The cascades are clearly different from UP/DOWN state transitions.^[^ ^49^, ^50^ ^]^ The UP/DOWN cortical states alternate at a much faster timescale (0.5–2 Hz),^[^ ^51^, ^52^, ^53^, ^54^ ^]^ whereas the cascades are infra‐slow (multiple seconds), brain‐wide sequential activations that unfold over much longer timescales and beyond cortical regions. Moreover, the UP phase is correlated positively with the activity of most neurons,^[^ ^55^ ^]^ whereas different phases of cascades involve the alternating activation of two distinct neuronal groups. Lastly, the UP/DOWN states are most prominent during non‐rapid eye movement (NREM) sleep and anesthetized conditions, whereas the cascades in the current study persist under stimulation. Nevertheless, it is possible that the UP/DOWN state transitions might contribute to the changes in delta‐band (1‐4 Hz) activity across the cascade cycle, given their overlapped frequencies.

Finally, our results suggest that the locomotion‐related visual coding enhancement observed in previous studies^[^ ^5^, ^9^, ^15^ ^]^ is enabled by the accompanying highly aroused brain state, rather than the act of locomotion itself. In examining the initiation and termination of running bouts, we found that the associated brain state dynamics, rather than the actual locomotion, promoted a sustained period of high visual encoding accuracy with hippocampal ripples being virtually absent. The locomotion‐related state change thus appeared to replace the intrinsic cascade dynamics with a continuously high arousal phase that prioritizes information encoding. Our findings may also be consistent with previous work suggesting that relatively low forebrain arousal is a prerequisite for optimal memory function.^[^ ^56^, ^57^ ^]^ During sustained periods of high arousal, such as locomotion, the brain has diminished internal mnemonic processes and thus deteriorated memory function compared to stationary modes of wakefulness. It is interesting to consider that memory consolidation, rather than being relegated to offline processing during sleep, is an active and prominent feature of the awake brain, but only during some behavioral states. From this perspective, the brain‐wide spiking cascades investigated in the present study may be central mediators of this process, periodically switching between endogenous cycles of sensory sampling and mnemonic engagement.

## 4 Experimental Section

### Neuropixels Data and Spike Sorting

The present study conducted data analysis utilizing the Brain Observatory Neuropixel dataset obtained from the Allen Institute.^[^ ^32^, ^36^ ^]^ This dataset comprises high‐density extracellular neuron recordings of mice using Neuropixel probes. Each mouse was implanted up to six Neuropixel probes, which targeted the primary visual cortex (VISp) and five high‐order visual cortical areas, namely, latero‐medial area (VISl), antero‐lateral area (VISal), rostro‐lateral area (VISrl), postero‐medial area (VISpm), and antero‐medial area (VISam). The silicon probes were inserted to a depth of up to 3.5 mm into the brain, enabling the recording of spiking activity within two visual thalamic nuclei, i.e., the lateral posterior nucleus (LP) and the lateral geniculate nucleus (LGN), as well as other regions that the probes traversed, such as the hippocampus (Figure 1A and Figure 3A).

It was focused on the experiments from all 32 mice performed with the “Brain Observatory 1.1” stimulus set containing various types of visual stimuli as shown in Figure S1A (Supporting Information). It was specifically narrowed the analysis to two types of stimuli: natural images and drifting gratings. During each experiment, the natural image and drifting grating stimuli were presented separately in three distinct sessions. Regarding the natural image stimuli, a continuous sequence of images was presented in each session, with each image maintained for a duration of 250 ms. Across the entirety of the experiment, a total of 118 distinct image stimuli were employed, each being repeated 50 times and randomly delivered throughout the three sessions (as illustrated in Figure 1B). For the drifting gratings, stimuli were displayed with a spatial frequency of 0.04 cycles/deg, 80% contrast, 8 directions (0°, 30°, 60°, 90°, 120°, 150°) and 5 temporal frequencies (1, 2, 4, 8, and 15 Hz). Across all three drifting grating sessions, each condition was repeated 15 times.

Processed neural spike data from the Allen Neuropixel dataset were utilized in this analysis. The neural spike data were sorted with the Kilosort2 pipeline (Stringer et al., 2019). Artefactual units are automatically filtered based on spread (single or >25 channels), shape (no clear peak/trough), and multiple spatial peaks. The automated process removes 94% of noise units (26% of the total), followed by manual inspection to eliminate the remaining noise. Further information regarding the processing of the spike data can be found in the white paper of the dataset.

### Mice Exclusion

It was did not confine the analysis to a small subset of mice; rather, distinct subsets were employed for various analyses to fully exploit the dataset. Initially, three mice were excluded due to insufficient stationary periods during task sessions (less than 10% of the time), resulting in a remaining cohort of 29 mice.

For the pupil size analysis, six mice lacking pupil data were excluded, yielding a cohort of 23 mice for the analysis. In the natural scene visual stimuli analysis, the objective was to compare decoding accuracy across three conditions: stationary‐high, stationary‐low, and running periods. Given a total of 5900 samples for 118 images with 50 repeats, mice with an insufficient number of samples (n ≤ 150) for each condition were excluded, leading to the removal of nine mice and leaving 20 mice for analysis. In the drifting‐gratings analysis, aimed at comparing the response of positive‐responding and negative‐responding neurons across three conditions, one mouse lacking stationary periods during the drifting‐grating session was excluded. This resulted in a final cohort of 28 mice included in the analysis. Removal details were shown in Table S2 (Supporting Information).

### Spiking Cascade Detection

Processed neural spike data from the Allen Neuropixel dataset were utilized in our analysis. The neural spike data were sorted with the Kilosort2 pipeline.^[^ ^30^ ^]^ Further information regarding the processing of the spike data could be found in the white paper of the dataset.

Following prior studies^[^ ^31^, ^48^ ^]^ to identify spiking cascade events, we first divided time evenly into 200 ms time bins and calculated spike rates by counting the number of spikes within each bin. Then a delay‐profile decomposition method was applied to extract the relative temporal relationship among a group of neurons in a data‐driven manner. Briefly, it was identified candidate neural events by segmenting the spiking rate data based on the troughs of the filtered global mean spiking rate (low‐pass, 0.25 Hz). For each neural event k, a delay profile vector **d**_*k*_ representing the temporal phase of each neuron within that event is derived.

(1) $$d_{k}={\left(t_{1k},\;t_{2k},\text{…},t_{\mathrm{Nk}}\right)}^{T}$$

where *t_ik_* representing the temporal centroid of the firing rate within the neural event k and N represent the total number of neurons in the recording. A delay profile matrix ***D*** representing all of the potential temporal relationships among the neurons can thus be constructed by combining the delay profiles for all M candidate neural events.

(2) $$D=\left(\begin{array}{cccc} d_{1} & d_{2} & \cdots & d_{M} \end{array}\right)=\left(\begin{array}{cccc} t_{11} & t_{12} & \cdots & t_{1M}\\ t_{21} & t_{22} & \cdots & t_{2M}\\ \vdots & \vdots & \cdots & \vdots \\ t_{N1} & t_{N2} & \cdots & t_{\mathrm{NM}} \end{array}\right)$$

Singular value decomposition (SVD) was then applied to the delay matrix ***D*** with the principal component u* defined as the principal delay profile representing the major sequential organization among the group of recorded neurons. It was then used the principal delay profile as a template to match the delay profile of candidate events. A candidate event that had a significant similarity score (*p* < 0.001) measured with Pearson's correlation was considered as a cascade event in this study.

### Positive‐Delay and Negative‐Delay Neurons

In the context of a cascade cycle, it was evident that the behavior of neurons was contingent upon their specific activation phase. In light of this observation, it was considered two discrete categories of neurons based on their activation phase within the spiking cascade cycle. These categories were denoted as the negative‐delay neurons (depicted by purple symbols in Figure 1C) and the positive‐delay neurons (represented by green symbols in Figure 1C).

The negative‐delay neurons exhibited a gradual recruitment during the initial phase of the cascade, followed by a rapid transition to the subsequent activation of positive‐delay neurons. The latter were subsequently discharged in a sequential manner during the later phase of the cascade. Formally, the negative‐delay neurons N and positive‐delay neurons P were defined as those whose mean delay value across all the candidate events, was significantly (*p* < 0.001) lower or higher than zero respectively.

### State Index Definition

Given the prominent role of the two neuron populations in regulating brain states (Figure 1C; Figure S4, Supporting Information), a state index s(t) by quantifying their relative activities for each time t was derived:

(3) $$s\;\left(t\right)=\gamma \;\bar{f_{P}\left(t\right)}-\left(1-\gamma \right)\bar{f_{N}\left(t\right)}\;\mathrm{with}\;\gamma =\frac{\sigma_{N}}{\sigma_{N}+\sigma_{P}}$$

where $\bar{f_{P}\left(t\right)}$, $\bar{f_{N}\left(t\right)}$, $\sigma_{P}$, $\sigma_{N}$ represent the mean firing rate of positive‐delay neurons, negative‐delay neurons, and the standard deviation of the mean firing rate of positive‐delay neurons and negative‐delay neurons respectively.

The distribution of the state index exhibits a bimodal pattern, as shown in Figure 1E. Consequently, a two‐class Gaussian mixture model was utilized to accurately model this bimodal distribution of the state index. The separation of these modes was achieved through the application of a threshold that corresponds to the optimal decision boundary. Specifically, periods characterized by state index values exceeding the threshold were categorized as stationary‐high states, while periods featuring state index values below the threshold were designated as stationary‐low states.

### Neural Population Decoding Analysis

To assess the sensory information encoded within the neuron population, we conducted an analysis of spiking data using nature scene image stimulation. In this protocol, a sequence of images was presented continuously, with each image stimulus having a duration of 250 ms. For each image stimulus, the neural code as a vector in which each entry corresponds to the count of spikes from a specific neuron was defined, measured within a 200 ms time window following the onset of the stimulus. For example, the neural code for the i‐th stimulus is:

(4) $$SR_{i}=\left(\begin{array}{cccc} f_{1i} & f_{2i} & \cdots & f_{\mathrm{Ni}} \end{array}\right)$$

where *f_ki_* denotes the k‐th neuron spike count within a 200 ms period following the i‐th stimulus onset.

It was then developed a method to quantify the degree of visual information encoded in the population neural code for each stimulus. The image stimuli were shuffled and divided into five groups, ensuring equitable distribution of stimuli classes. Within each group, there were 10 repetitions for every class, and special attention was given to prevent any overlap of stimuli across these groups. For each iterative step, one group was designated as the test dataset, while the remaining four groups served as training data. In the training phase of each iteration, a Support Vector Machine (SVM)‐based decoder was employed. This decoder was trained to establish a link between the population neural codes and the corresponding stimulus classes. Subsequently, this trained decoder was employed to make predictions on the test data. This process was repeated for all five iterations, and as a result, each stimulus was assessed and categorized as either correctly classified or misclassified. The overall decoding accuracy was computed as the average rate of accurate classification across all stimuli:

(5) $$\mathrm{acc}=\frac{1}{n}\;\sum_{k=1}^{n}C_{k}\;\mathrm{with}\;C_{k}=\left\{\begin{array}{cc} 1 & \mathrm{if}\;k\;\mathrm{is}\;\mathrm{correctly}\;\mathrm{classified}\\ 0 & \mathrm{if}\;k\;\mathrm{is}\;\mathrm{misclassified}, \end{array}\right.$$

where n is the number of stimuli in consideration. In this manner, the decoding accuracy can be evaluated for different states or conditions, such as immobile and locomotion states.

In addition to natural scene image stimuli, the same decoding analysis was also conducted for drifting‐grating stimuli to estimate stimulus direction and temporal frequency (Figure S14, Supporting Information). To validate the robustness of the analysis, various decoder models was examined, i.e., logistic regression (Figure S9, Supporting Information) and three‐layer multilayer perceptron with 512 hidden units (Figure S10, Supporting Information). To validate the integrity of this methodology, a control analysis using the exact same approach was conducted. The only distinction was that the stimulus identities (labels) randomly was shuffled, as shown in Figure S13 (Supporting Information). This control analysis helped us ascertain the robustness of this results.

To evaluate the impact of the state index on decoding accuracy, as shown in Figure 2C and Figure S7A (Supporting Information), trials were organized based on their state index values into groups. These groups were formed by segmenting the state index into bins with a step size of 0.1. For each group, the decoding accuracy was calculated as the mean accuracy of all trials belonging to that group.

To analyze the modulation of the decoding accuracy across the cascade cycle, as shown in Figure 2E, it was constructed time series of decoding accuracy *f_acc_* (*t*) for each mouse defined as:

(6) $$f_{\mathrm{acc}}\left(t\right)=\sum_{k=1}^{n}\;f_{\mathrm{acc}}^{\left(k\right)}\left(t\right)\mathrm{with}\;f_{\mathrm{acc}}^{\left(k\right)}\left(t\right)=\left\{\begin{array}{cc} C_{k} & \mathrm{if}\;T_{k}\leq t\leq T_{k}+\Delta T\\ \mathrm{NaN} & \text{if k is misclassified}, \end{array}\right.$$

where T_k_ is the onset time of stimulus for the k‐th trial, and Δ*T* is the time window following the stimulus onset, which is set to 0.2 seconds throughout our study. The NaN stands for “Not a Number” and is omitted in the summation in the equation.

### Neuron Evoked Responses Analysis

It was analyzed single neuron encoding of external stimuli with spiking data from drifting‐grating sessions as it contains a 1 s baseline period before every stimulus, providing suitable noise control. Drifting‐grating responding neurons were defined as those exhibiting a statistically significant difference in spiking rate during the stimulation period (0–600 ms post stimulus onset) compared to the baseline period (−800–0 ms), as determined by a paired *t*‐test with a significance level of p<0.001.

These drifting‐grating responding neurons could either be significantly enhanced or suppressed by the presented stimuli. Accordingly, it was classified neurons as “enhanced” or “suppressed” based on whether they displayed an increase or decrease in spiking rate, respectively, during stimulus presentation.

It was next investigated how the pre‐stimulus brain state affects single neuron response. The neuron evoked response was quantified by the spiking rate difference between the stimulation onset (0–400 ms) and baseline periods (−800–0 ms). As single neuron activity might be confounded by the ongoing state‐dependent dynamics, it was focused on the drifting‐grating responding neurons without those significantly modulated by the cascade, i.e., the positive‐delay and negative‐delay neurons (Figure S15B, Supporting Information). The neural responses elicited by each stimulus separately for both enhanced neurons and suppressed neurons are defined as:

(7) $$e_{E_{i}}^{i}=\frac{1}{\left|E_{i}\right|}\;\sum_{k\in E_{i}}f_{k}^{i}\;e_{S_{i}}^{i}=\frac{1}{\left|S_{i}\right|}\;\sum_{k\in S_{i}}f_{k}^{i}\;$$

where $E_{i}$, $S_{i}$ represent the set of enhanced neurons and suppressed neurons of the i‐th stimulus, $e_{E_{i}}^{i}$, $e_{S_{i}}^{i}$ represent the evoked response for i‐th stimulus from the two neuron sets, and | · | denotes the size of the set. The pre‐stimulus state was defined as the state index during the 200 ms pre‐stimulus period.

### Local Field Potential Analysis

Delta power and gamma power were computed for local field potentials (LFPs) across all recorded channels. To calculate delta power, a band‐pass filter (1‐4 Hz) was applied to the LFP signal of each channel, followed by rectification and lowpass filtering (<0.72 Hz, corresponding to π cycles of the mean band‐pass frequencies). Similarly, gamma power was extracted using a comparable procedure, with the exception of applying a band‐pass filter (55–65 Hz) and setting the low‐pass filter cutoff frequency to 19 Hz with respect to π cycles.

Hippocampal sharp wave ripples (SWRs) are brief, high‐frequency oscillations (110‐200 Hz) that can be observed in the local field potential (LFP) recorded from hippocampal recording sites. For ripple detection in this study, it was employed an offline method^[^ ^31^, ^58^ ^]^ utilizing the LFP signal (1250 Hz) captured from the hippocampal CA1 region. The identification of ripple events was conducted individually for each CA1 recording site (channel), resulting in robust and extensively overlapping ripple detection across the channels. To consolidate the detection outcomes from various channels, a criterion was imposed: a detected ripple event was deemed valid only if it was identified in more than 40% of the CA1 channels.

### Behavior Data

*Stationary and Running State*: Throughout the recording sessions, the mice exhibited significant amounts of both running and stationary periods. To delineate these periods, a low‐pass filter (a 3rd‐order Butterworth filter with a cutoff frequency set at 2 Hz) to the running speed data extracted from the dataset was first applied. This filtration aimed to eliminate high‐frequency noise. Negative running speeds were attributed solely to sensor noise.^[^ ^31^ ^]^ Consequently, a threshold was determined at the 0.05 percentile of the absolute values of the negatively filtered speed values. Any time point at which the filtered running speed exceeded this threshold was categorized as a non‐stationary point.

Given that short stationary periods were interspersed with non‐stationary periods, this selection process focused exclusively on stationary periods that persisted for a duration exceeding 20 s. Additionally, these stationary periods were required to have a minimum gap of 3 s between identified stationary and non‐stationary periods to mitigate potential boundary effects. Similarly, running periods were identified using the same approach, but the selection was confined to non‐stationary periods lasting longer than 0.5 s and a minimum time gap of 3 s was mandated between stationary and running periods.

### Pupil Size

The eye‐tracking data from the dataset were pre‐processed with a set of parameters pre‐computed at a sampling rate of 30 Hz. We used pupil diameter as the arousal index in our analysis and it was defined as the mean of the pupil height and width. To eliminate high‐frequency noise, the pupil diameters were subjected to low‐pass filtering with a cutoff frequency set at 2 Hz.

### Modulation Across Spiking Cascade Cycles

We examined variations in signals, such as decoding accuracy, hippocampal ripple rates, and pupil size, across the cycle of spiking cascades. The spiking cascades were characterized by a marked increase in the activity of positive‐delay neurons at their midpoint. Following the previous definition,^[^ ^31^ ^]^ it was identified these moments as the local peak of the first‐order temporal derivative of the mean spiking time course of the positive‐delay neurons. To evaluate the modulation of these various signals throughout the cascade cycle, it was aligned and averaged these time‐series signals relative to the midpoint's timing across all detected cascade cycles for each mouse.

### Statistical Analysis

A two‐sided one‐sample *t*‐test was conducted to evaluate differences in pupil size across state conditions (Figure 1F; Figure 4D and *N* = 26) and to assess variations in response amplitude between state conditions (Figure 5D and *N* = 28). A two‐sided paired *t*‐test was employed to compare decoding accuracy across different state conditions (Figure 2B; Figure 4E and *N* = 20). Group differences in hippocampal SPW‐R rates between state conditions (Figure 4B) were assessed using a two‐sided two‐sample *t*‐test. Additionally, Pearson correlation was applied to examine the linear relationship between principal delay profiles across conditions (Figure S1B, Supporting Information) and between the state index and decoding accuracy (Figure 2C; Figure 3D).

For all statistical analyses, a p‐value of less than 0.05 was considered statistically significant. The following symbols were used to indicate the degree of significance: ns for *p* > 0.05, * for *p* < 0.05, ** for *p* < 0.01, *** for *p* < 0.001, and **** for *p* < 0.00001.

It was used the Neuropixels Visual Coding dataset from the Allen Institute.^[^ ^32^, ^36^ ^]^ All the multimodal data were available at https://portal.brain‐map.org/explore/circuits/visual‐coding‐neuropixels. The Python code that produced the major results of this paper will be available at https://github.com/psu‐mcnl/Neural‐Arousal.

## Conflict of Interest

The authors declare no conflict of interest.

## Author Contributions

Y.Y. and X.L. contributed to the conception, design of the work, and data analysis; X.L. also devoted the efforts to the supervision, project administration and funding acquisition; Y.Y., D.A.L., J.H.D. G.O.S. and X.L. contributed to data visualization, and writing the paper.

## Supporting information



Supporting Information

## Data Availability

The data and code that produced the major results of this paper will be available at https://github.com/psu‐mcnl/Neural‐Arousal.
