# Supplementary material for: Sensory Encoding Alternates With Hippocampal Ripples across Cycles of Forebrain Spiking Cascades
Source: Adv Sci (Weinh). 2025 Feb 27;12(16):2406224. doi: 10.1002/advs.202406224 (PMC12021030; doi:10.1002/advs.202406224)
Supplement: Supplementary file 1 — Supporting Information [file ADVS-12-2406224-s001.pdf]

ADVANCED SCIENCE
Open Access

Supporting Information

for *Adv. Sci.*, DOI 10.1002/advs.202406224

Sensory Encoding Alternates With Hippocampal Ripples across Cycles of Forebrain Spiking Cascades

*Yifan Yang, David A. Leopold, Jeff H. Duyn, Grayson O. Sipe and Xiao Liu**

Supplementary Information for Sensory encoding alternates with hippocampal ripples across cycles of forebrain spiking cascades

Yifan Yang[1], David A. Leopold[3,4], Jeff H. Duyn[5], Grayson O. Sipe[6], Xiao Liu[1,2*]

[1]Department of Biomedical Engineering, The Pennsylvania State University, University Park, 16802, PA, USA.
[2]Institute for Computational and Data Sciences, The Pennsylvania State University, University Park, 16802, PA, USA.
[3]Neurophysiology Imaging Facility, National Institute of Mental Health, National Institute of Neurological. Disorders and Stroke, and National Eye Institute, National Institutes of Health, Bethesda, 20892, MD, USA.
[4]Section on Cognitive Neurophysiology and Imaging, Laboratory of Neuropsychology, National Institute of Mental Health, National Institutes of Health, Bethesda, 20892, MD, USA.
[5]5Advanced MRI Section, Laboratory of Functional and Molecular Imaging, National Institute of Neurological Disorders and Stroke, National Institutes of Health, Bethesda, 20892, MD, USA.
[6]5Department of Biology, The Pennsylvania State University, University Park, 16802, PA, USA.

*Corresponding author(s). E-mail(s): xx1213@psu.edu;
Contributing authors: yzy161@psu.edu; leopoldd@mail.nih.gov; jeff.duyn@nih.gov; gsipe@psu.edu;

This PDF file includes:

- Tables S1 to S3
- Figures S1 to S15

Contents

Page

1 Supplementary Tables **3**
1.1 Table S1 3
1.2 Table S2 4
1.3 Table S3 5

2 Supplementary Figures **6**
2.1 Figure S1 6

2.2 Figure S2 7
2.3 Figure S3 8
2.4 Figure S4 9
2.5 Figure S5 10
2.6 Figure S6 11
2.7 Figure S7 12
2.8 Figure S8 13
2.9 Figure S9 14
2.10 Figure S10 15
2.11 Figure S11 16
2.12 Figure S12 17
2.13 Figure S13 18
2.14 Figure S14 19
2.15 Figure S15 20

1 Supplementary Tables

1.1 Table S1

Summary of key findings evident sensory response enhancement

Sources of Enhanced Sensory Responses	***Examples***	***References***
Active Behaviors	locomotion, whisking	Gentet et al., 2010; Niell and Stryker, 2010; Saleem et al., 2013; Zagha et al., 2013; Christensen and Pillow, 2022; Renart et al., 2010a; Bennett et al., 2013; Ecker et al., 2014; Musall et al., 2019; McCormick et al., 2020
Induced Arousal Modulations	air puff	Vinck et al., 2015
	noradrenergic and cholinergic interventions	Lee and Dan, 2012; Pinto et al., 2013; Polack et al., 2013; Fu et al., 2014; Lee et al., 2014
Multi-second Spontaneous Arousal Modulations	ongoing brain activity	Fox et al., 2006; He, 2013;
	spontaneous pupil fluctuations	Reimer et al., 2014; Reimer et al., 2016; McGinley et al., 2015a, 2015b

1.2 Table S2

Mice exclusion details.

	# of Mice removed	# of Mice remained	Removal details
Initial removal	3	29	Mice with insufficient stationary periods (≤10%) were removed.<br>• 758798717: 0.3% stationary periods<br>• 760693773: 1.8% stationary periods<br>• 762602078: 0.0% stationary periods
Pupil analysis	6	23	Mice with no pupil data available were removed.<br>• 715093703<br>• 719161530<br>• 721123822<br>• 732592105<br>• 737581020<br>• 739448407
Natural scene visual stimuli decoding analysis	9	20	Mice with insufficient number of samples (n≤150) for any of the condition (stationary-high, stationary-low, running) were excluded.<br>• 732592105: 76 samples for stationary-high.<br>• 737581020: 141 samples for stationary-high.<br>• 746083955: 81 samples for stationary-high.<br>• 757216464: 0 samples for both stationary-high and low.<br>• 760345702: 0 samples for both stationary-high and low.<br>• 761418226: 0 samples for both stationary-high and low.<br>• 762120172: 48 samples for stationary-high.<br>• 791319847: 127 samples for stationary-high.<br>• 798911424: 147 samples for stationary-low.
drifting-gratings response analysis	1	28	Mice lacking stationary periods during the drifting-grating sessions were excluded.<br>• 75134857: 0.0% stationary periods during drifting-grating sessions.

1.3 Table S3

Stationary/running periods summarized for stimulus sessions across all mice.

Sessions	Stationary periods (sec)	Stationary percentage (%)	Running periods (sec)	Running percentage (%)
Nature image	542.4 ± 457.2	44.3 ± 34.0	625.8 ± 409.3	55.7 ± 34.0
Drifting-gratings	863.5 ± 559.0	56.1 ± 31.1	622.3 ± 461.6	43.9 ± 31.1
Spontaneous	552.6 ± 321.8	55.3 ± 27.9	412.4 ± 254.8	44.7 ± 27.9

* Mean ± SD

2 Supplementary Figures

2.1 Figure S1

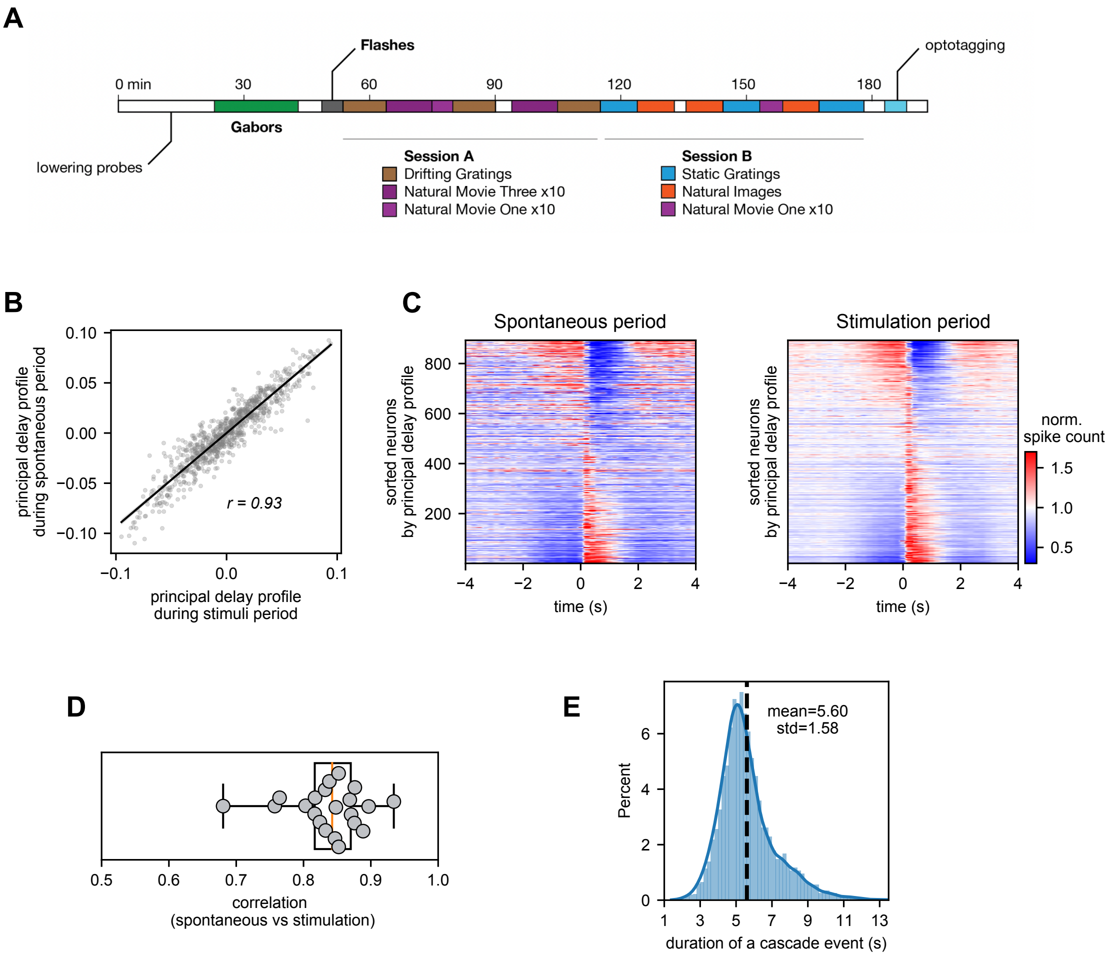

Figure S1. Spiking cascades pervade the cortex during both visual stimulation and rest. (A) Illustration of the "Brain Observatory" stimulus set from Allen Visual Coding - Neuropixel dataset. (B) Assessment of the similarity between the principal delay profile obtained from spontaneous neural activity and that from neural activity during visual stimulation in a representative mouse. The degree of similarity is quantified using Pearson's correlation coefficient. (C) The average pattern of the spiking cascade during spontaneous period (Left) and visual stimulation period (Right) from the representative mouse. (D) Box plot illustrating the correlation between principal delay profiles of spontaneous and visual stimulation sessions across individual mice. Each data point represents a distinct mouse. (E) The distribution summarizes the duration of all detected cascades across all mice. The dotted line represents the mean of the distribution.

2.2 Figure S2

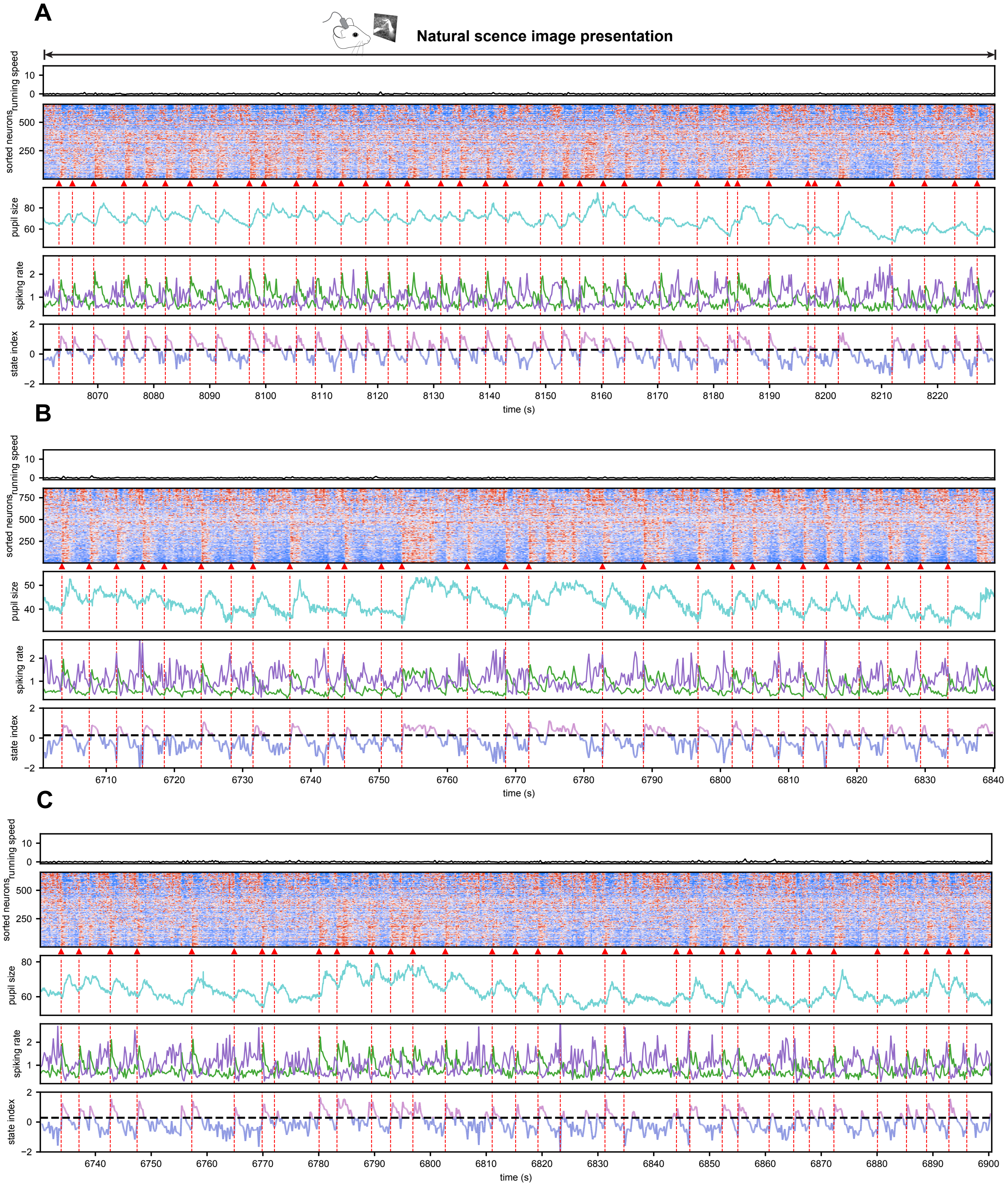

Figure S2. (A)−(C) Examples of spiking cascade during continuous natural scene image stimulation in the absence of running.

2.3 Figure S3

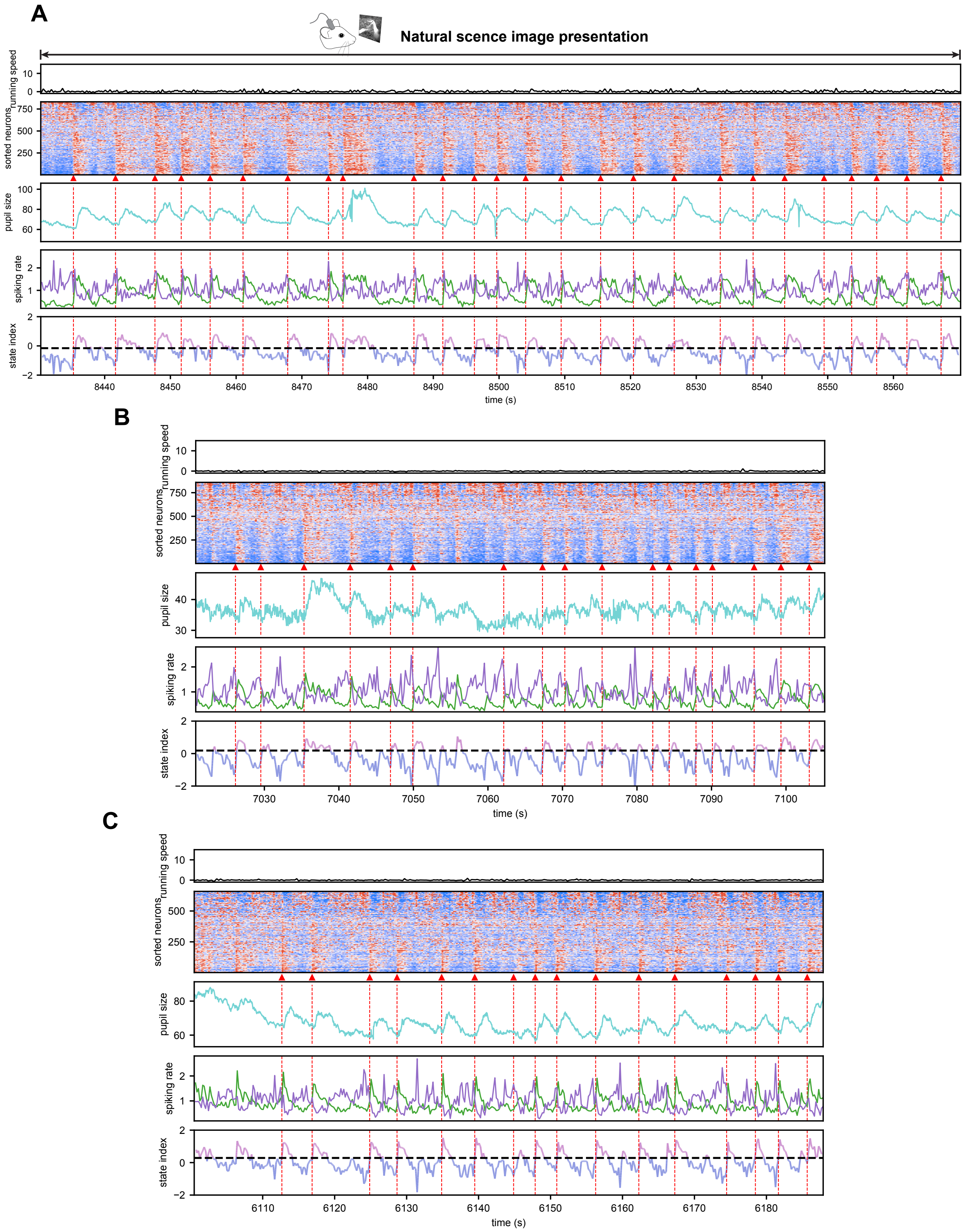

Figure S3. (A)–(C) Examples of spiking cascade during continuous natural scene image stimulation in the absence of running.

2.4 Figure S4

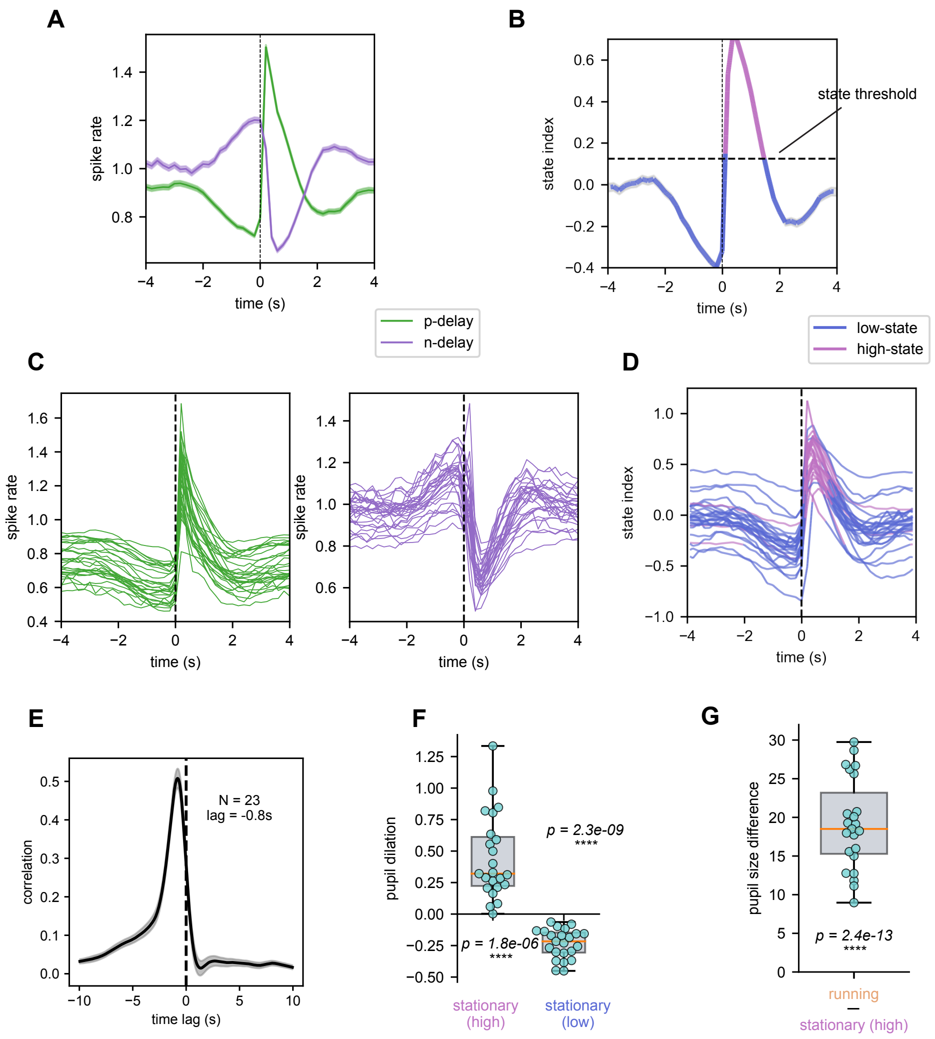

Figure S4. Spiking cascade is accompanied by seconds-scale arousal modulations. (A) Average activity of positive-delay neurons (green) and negative-delay neurons (purple) throughout the cascade cycle, shown for the representative mouse. (B) Averaged state index across the cascade cycle, illustrated for the representative mouse. The threshold distinguishing high and low states is indicated by a dashed line. (C) Average activity of positive-delay neurons (left, green) and negative-delay neurons (right, purple) throughout the cascade cycle across all mice. Each line represents a different mouse. (D) Averaged state index across the cascade cycle across all mice. Each line corresponds to a different mouse, and color denotes stationary high/low states. (E) Group-averaged cross-correlation between the state index and pupil size during stationary periods. (F) Box plot presenting pupil dilation for both stationary-high and stationary-low states. Each dot represents an individual mouse. (G) Box plot demonstrating the difference in pupil size between running state and stationary-high state, with each dot representing a distinct mouse.

2.5 Figure S5

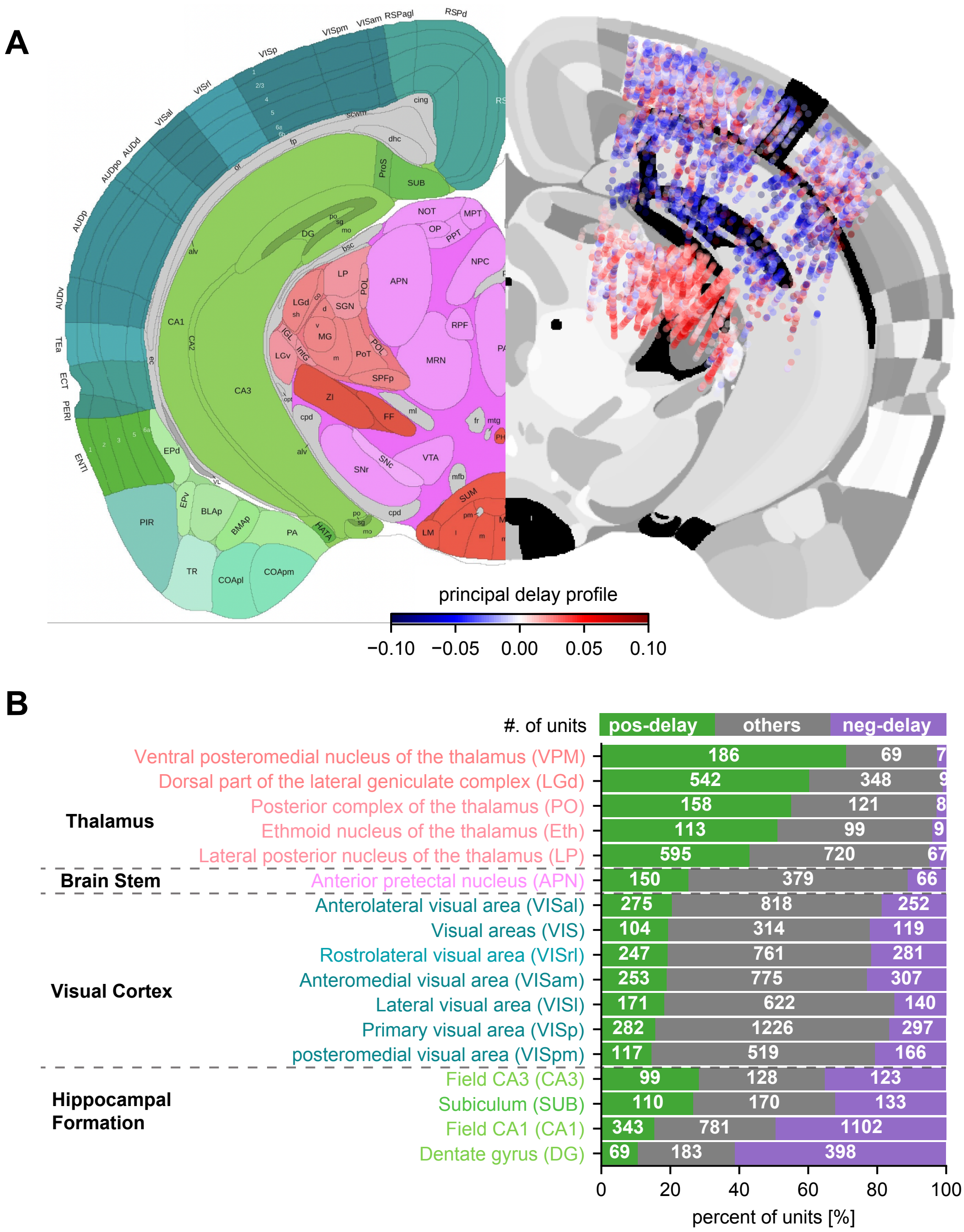

Figure S5. Spatial organization of neurons with negative and positive delays. (A) Spatial map of the principal delay profile values, where each dot represents a channel of the Neuropixels probe. The principal delay profile value is averaged for neurons detected by each channel. (B) Boxplot showing the region-specific distribution of the percentage of three neuron groups: positive-delay neurons (green), negative-delay neurons (purple), and other neurons (gray).

2.6 Figure S6

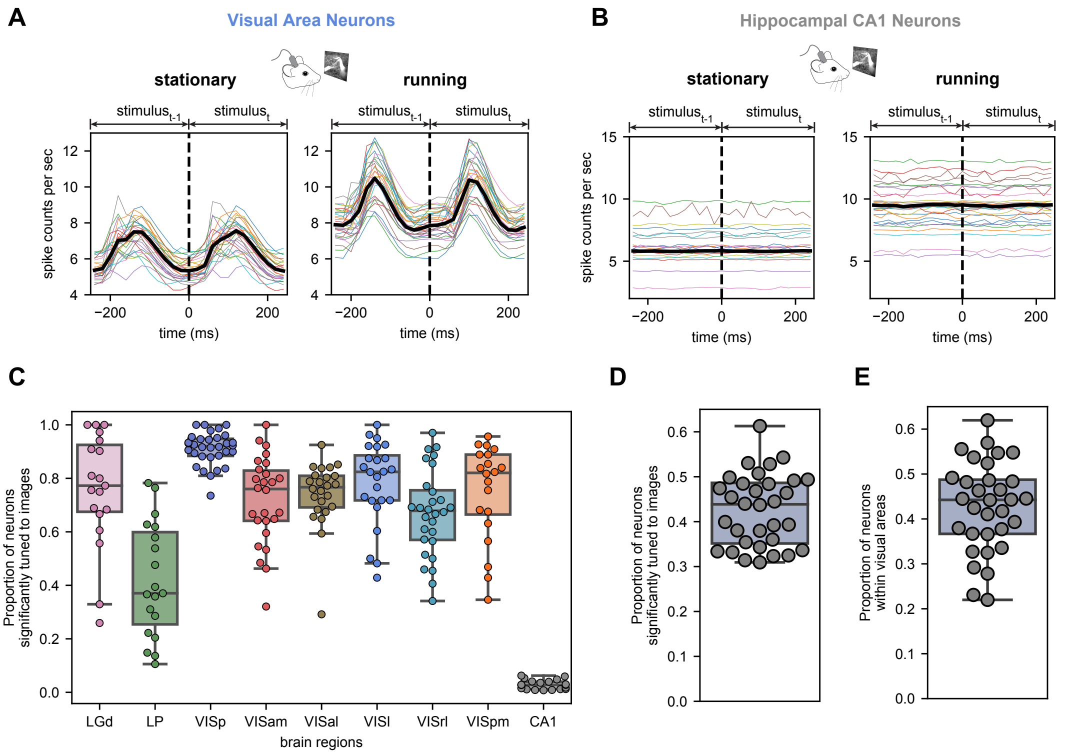

Figure S6. Neuronal responses to continuous visual stimuli. (A) Average activity of visual area neurons exhibiting tuning to continuous image stimuli during both stationary and running states. Each curve represents a distinct mouse. (B) In contrast, the average activity of hippocampal neurons remained unaffected by continuous image stimuli. (C) Box plot showing the proportion of neurons significantly tuned to image stimuli (image-tuned neurons) across different brain regions, with each data point representing an individual mouse. (D) Box plot illustrating the proportion of image-tuned neurons relative to all recorded neurons, with each dot representing a mouse. (E) Same as (D), but for neurons within visual cortical areas.

2.7 Figure S7

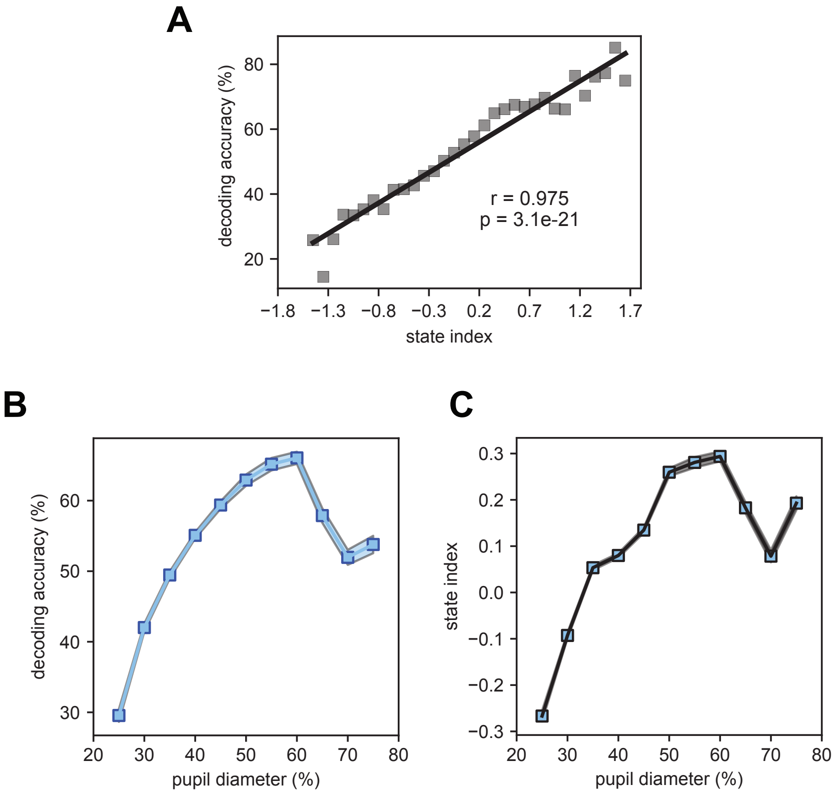

Figure S7. (A) Linear relationship between state index and decoding accuracy averaged across all the mice during stationary periods. The linear relationship is measured by Pearson's correlation. Pupil diameter as arousal indicator reveals an inverse-U association (B-C). (B) Group-averaged decoding accuracy and (C) state index both peak at an intermediate pupil diameter (60% of maximum). Shaded areas represent the standard error of the mean.

2.8 Figure S8

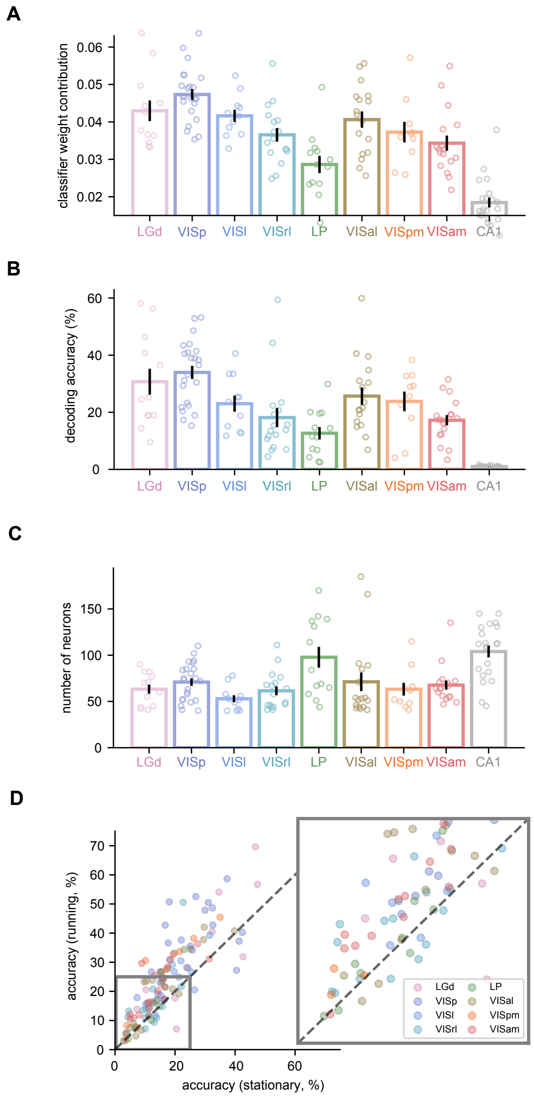

Figure S8. State dependent visual coding is evident in all visual areas. (A) Bar plot showing the averaged classifier weights importance of each brain region. Each dot represents a specific brain region in the corresponding mouse. (B) Bar plot illustrating the decoding accuracy of each brain region using data exclusively from that region. Each dot represents a region in the corresponding mouse. (C) Bar plot displaying the number of neurons in each region. Each dot represents a region in the corresponding mouse. (D) Changes in region-wise decoding accuracy between stationary and running conditions. Each colored dot represents a specific visual region from a single mouse.

2.9 Figure S9

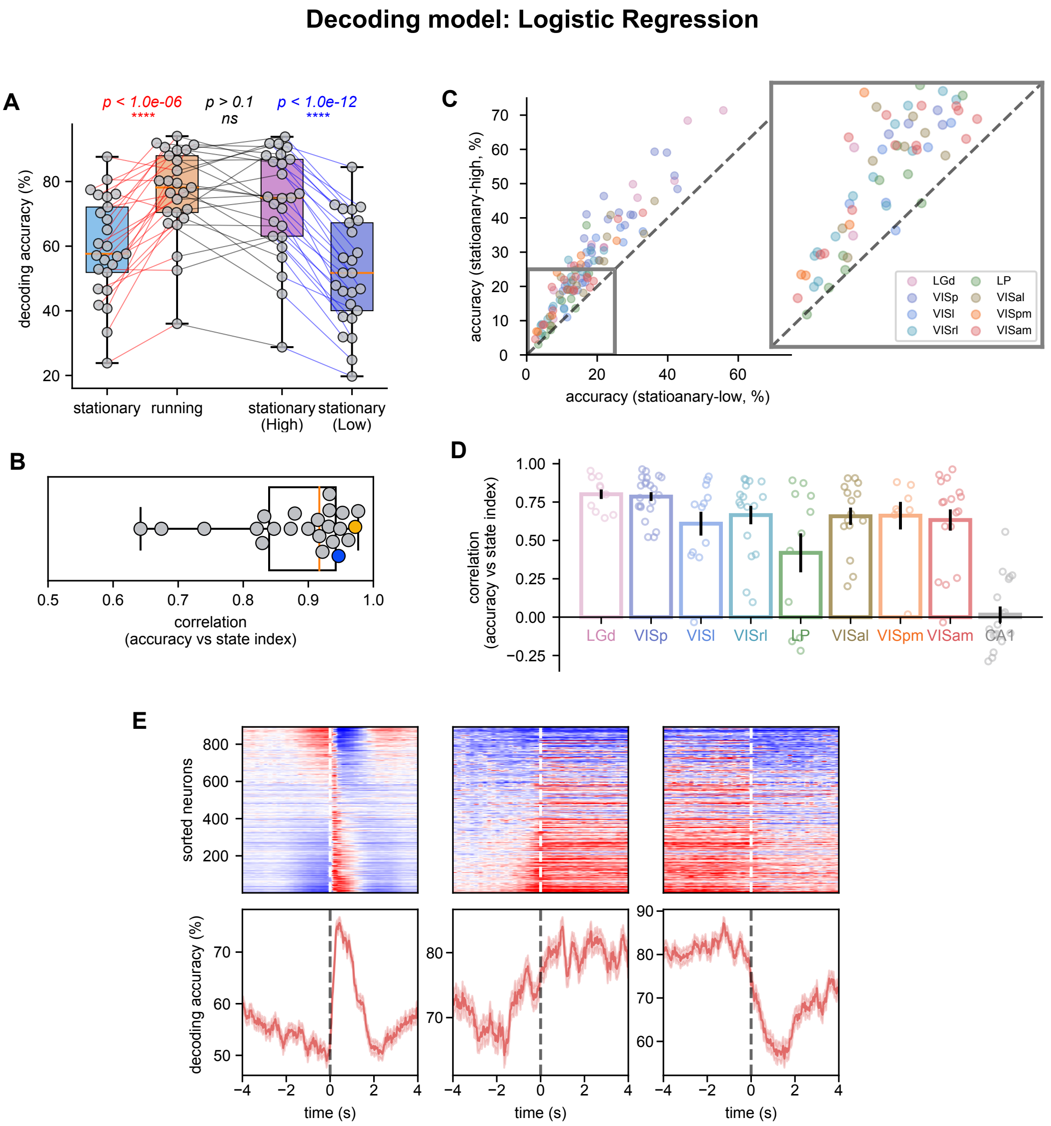

Figure S9. Decoding neural activities with logistic regression. (A) Box plot showing the decoding accuracy within-subject changes under different conditions. Each dot represents a mouse and pairwise t-test is used for significance test. (B) Linear relationship between state index and decoding accuracy is summarized in box plot for all mice where the yellow and blue dots represent the example mice correspondingly. The linear relationship is measured by Pearson's correlation. (C) Change in region-wise decoding accuracy between stationary-high and stationary-low state. Each colored dot represents visual region indicated by the color from a mouse. (D) Box plot showing the linear relationship between the state index and decoding accuracy for each brain region, similar to (C). Each dot represents a mouse with the corresponding region recorded. (E) Decoding accuracy across the 8-s cascade cycle (Left), running onset (middle) and offset (Right) from all 32 mice. Note the averaged cascade pattern is from the representative mice.

2.10 Figure S10

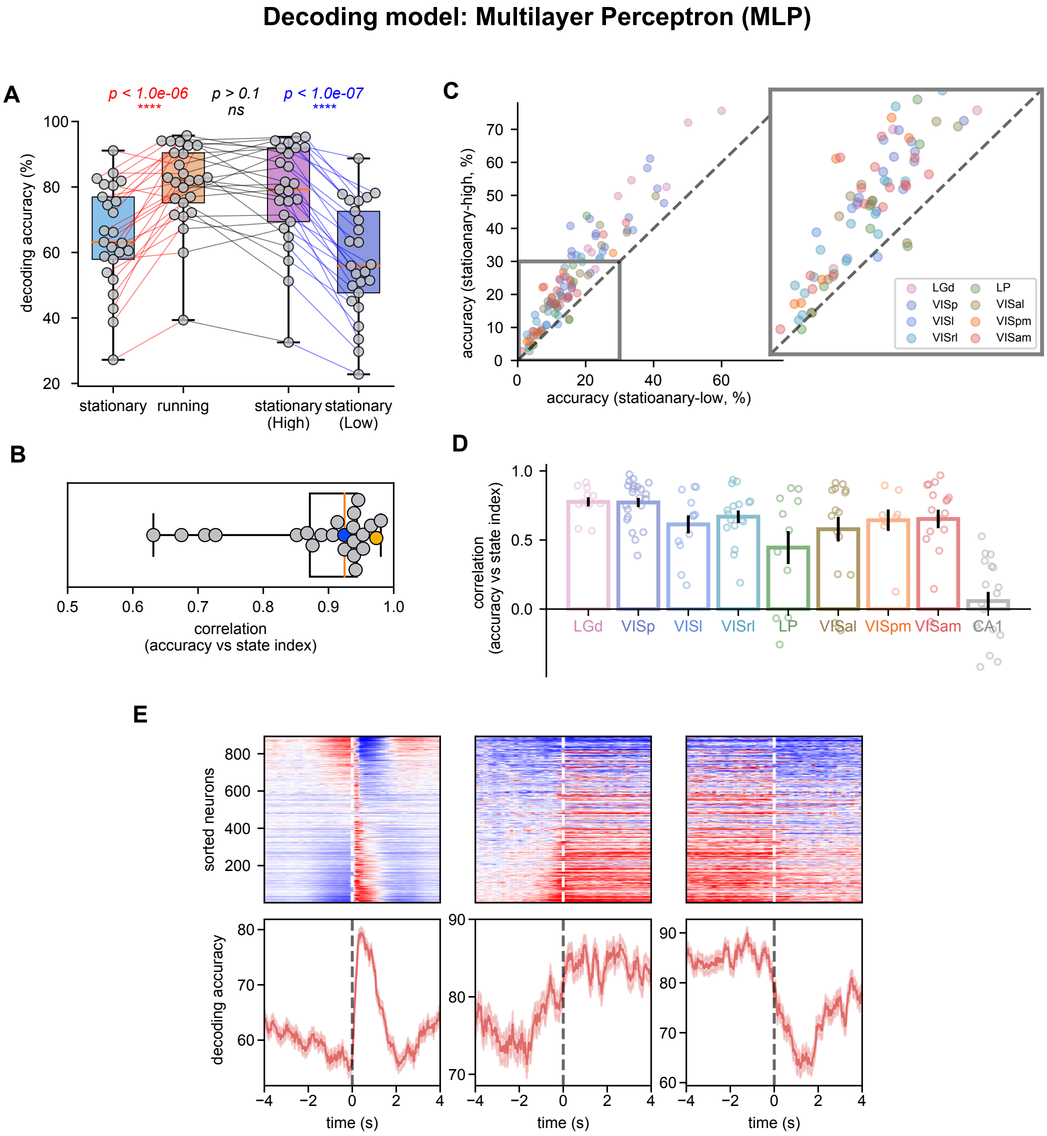

Figure S10. Decoding neural activities with multilayer perceptron (MLP). (A) Box plot showing the decoding accuracy within-subject changes under different conditions. Each dot represents a mouse and pairwise t-test is used for significance test. (B) Linear relationship between state index and decoding accuracy is summarized in box plot for all mice where the yellow and blue dots represent the example mice correspondingly. The linear relationship is measured by Pearson's correlation. (C) Change in region-wise decoding accuracy between stationary-high and stationary-low state. Each colored dot represents visual region indicated by the color from a mouse. (D) Box plot showing the linear relationship between the state index and decoding accuracy for each brain region, similar to (C). Each dot represents a mouse with the corresponding region recorded. (E) Decoding accuracy across the 8-s cascade cycle (Left), running onset (middle) and offset (Right) from all 32 mice. Note the averaged cascade pattern is from the representative mice.

2.11 Figure S11

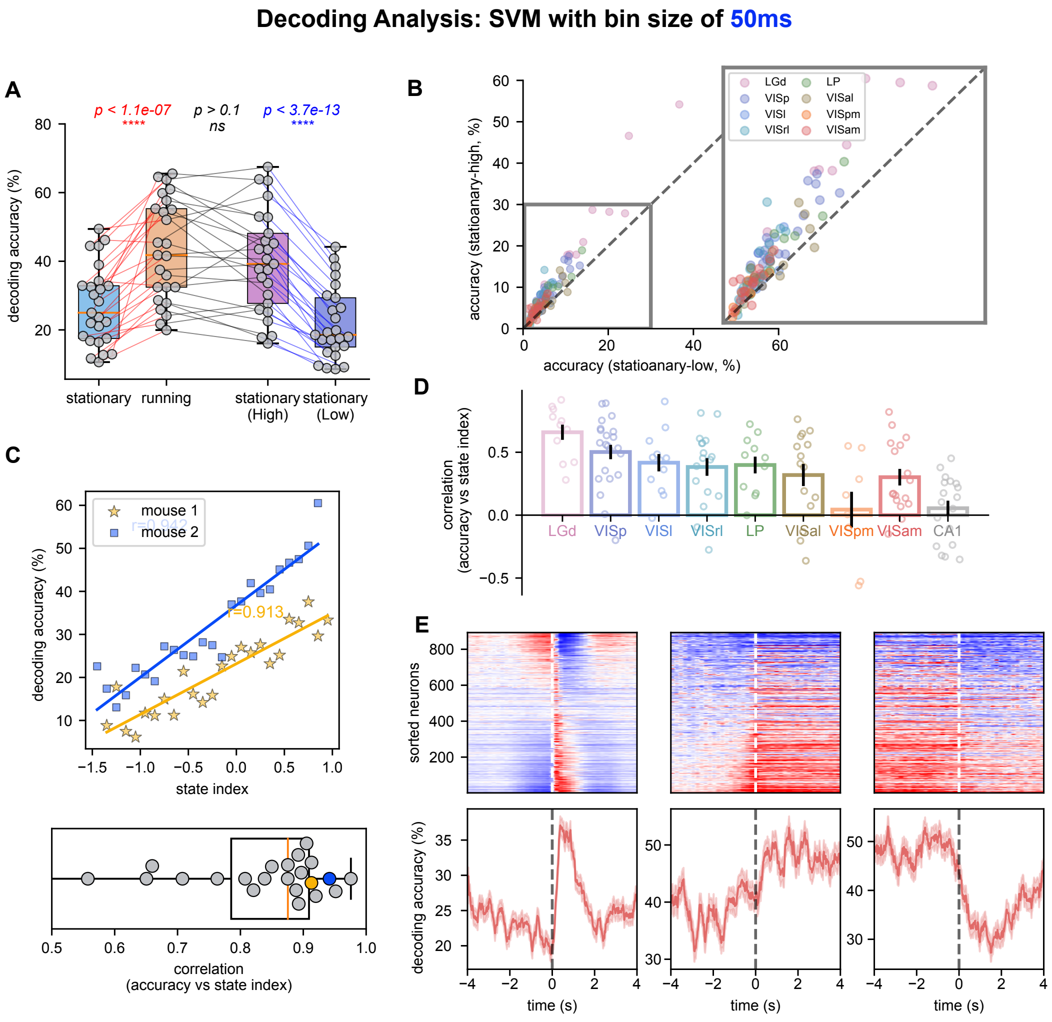

Figure S11. Decoding neural activities with time bin of 50ms. (A) Box plot showing the decoding accuracy within-subject changes under different conditions trained with shuffled stimuli labels. Each dot represents a mouse and pairwise t-test is used for significance test. (B) Bar plot showing the averaged classifier weights importance of each brain region with the classifier trained with shuffled stimuli labels. Each dot represents a region in the corresponding mouse.

2.12 Figure S12

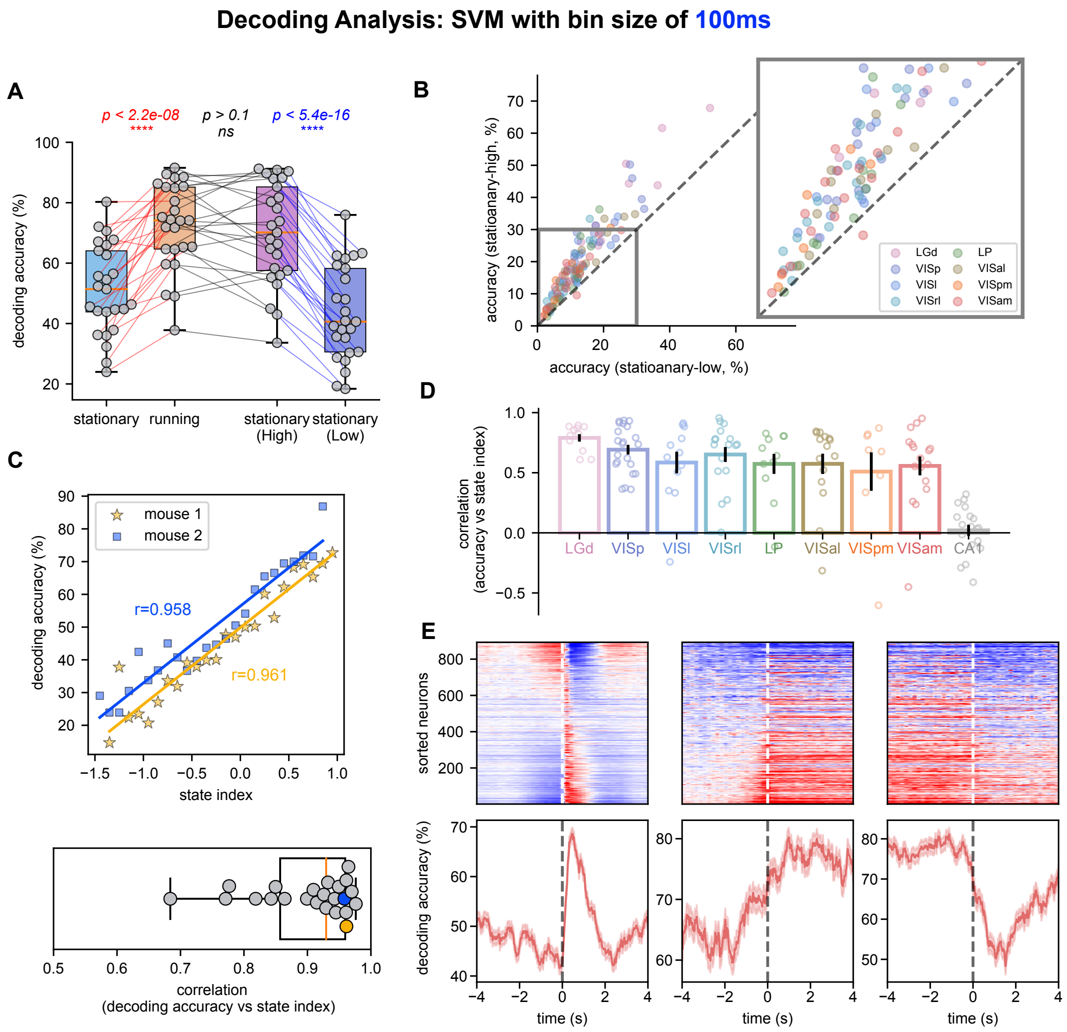

Figure S12. Decoding neural activities with time bin of 100ms. (A) Box plot showing the decoding accuracy within-subject changes under different conditions trained with shuffled stimuli labels. Each dot represents a mouse and pairwise t-test is used for significance test. (B) Bar plot showing the averaged classifier weights importance of each brain region with the classifier trained with shuffled stimuli labels. Each dot represents a region in the corresponding mouse.

2.13 Figure S13

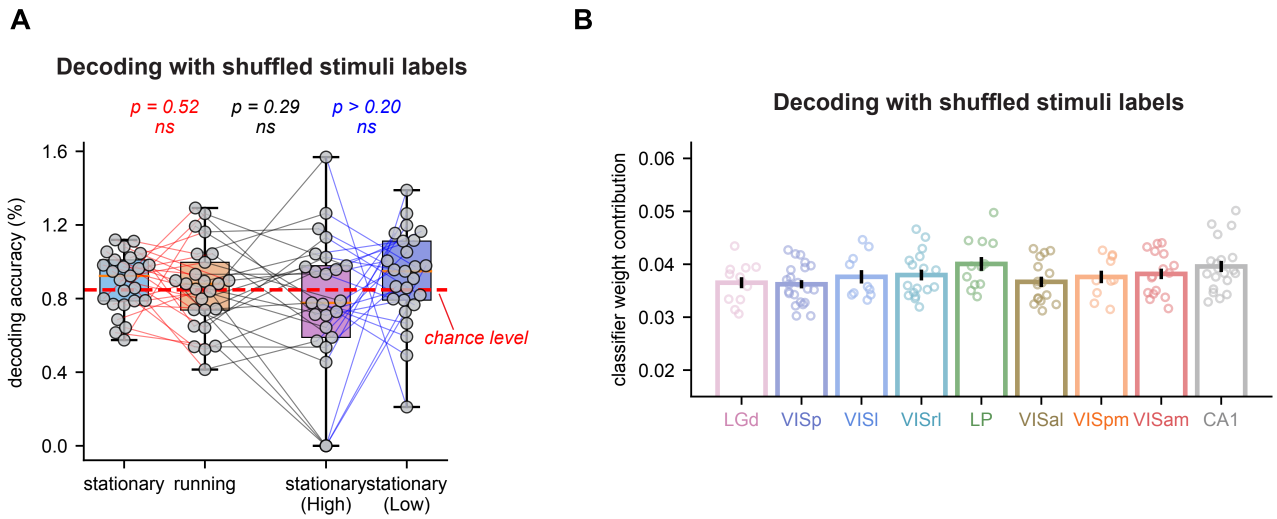

Figure S13. Decoding neural activities with shuffled stimuli labels. (A) Box plot showing the decoding accuracy within-subject changes under different conditions trained with shuffled stimuli labels. Each dot represents a mouse and pairwise t-test is used for significance test. (B) Bar plot showing the averaged classifier weights importance of each brain region with the classifier trained with shuffled stimuli labels. Each dot represents a region in the corresponding mouse.

2.14 Figure S14

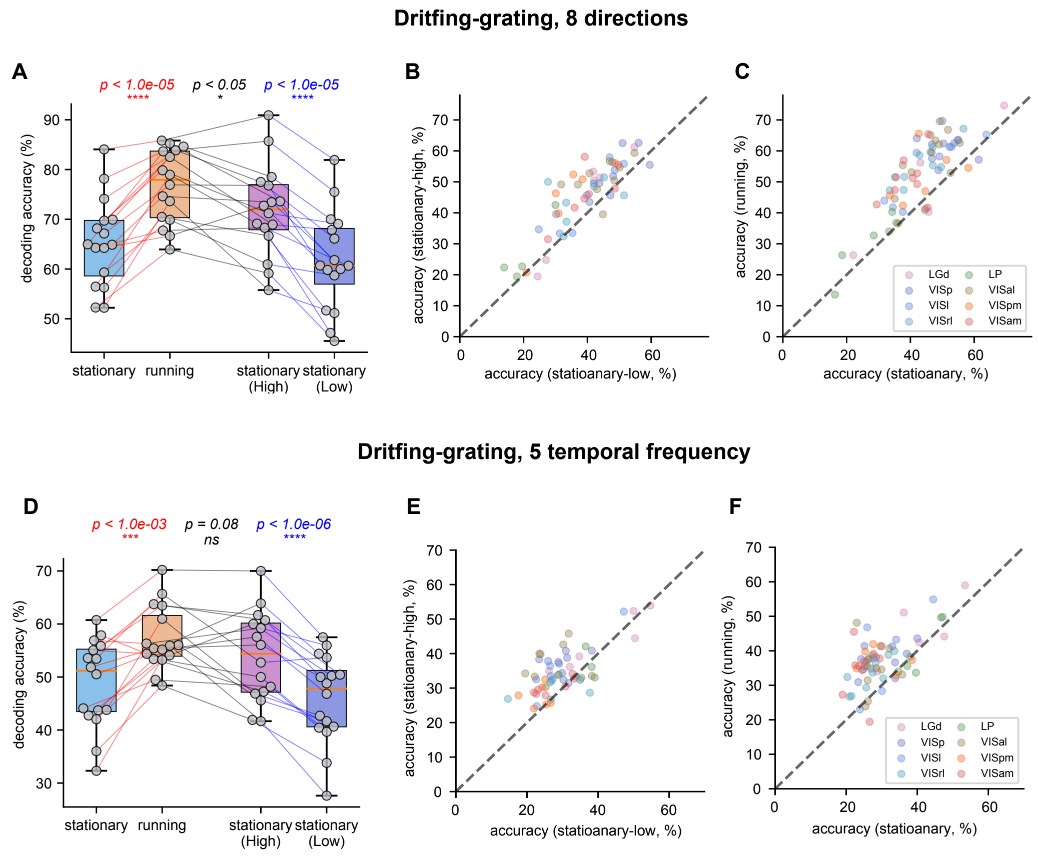

Figure S14. State-dependent population encoding of visual drifting-grating stimuli. (A) Box plot showing the accuracy of decoding drifting-grating direction within-subject changes under different conditions. Change in region-wise decoding accuracy of drifting-grating direction between stationary-high and stationary-low state (B) and between running and stationary (C). (D) Box plot showing the accuracy of decoding drifting-grating temporal frequency within-subject changes under different conditions. Change in region-wise decoding accuracy of drifting-grating temporal frequency between stationary-high and stationary-low state (E) and between running and stationary (F).

2.15 Figure S15

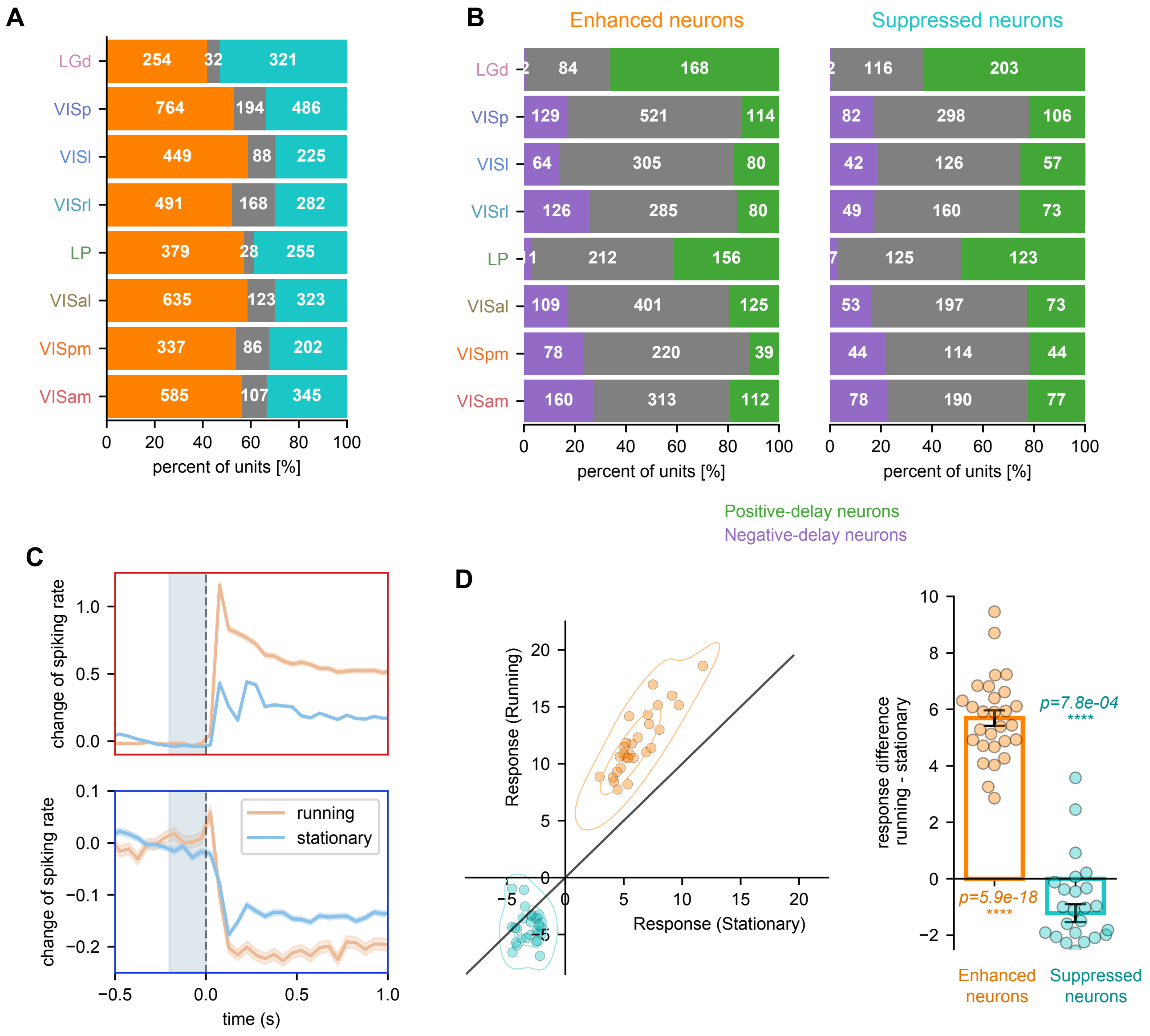

Figure S15. Single-neuron responses to Drifting-Grating stimuli. (A) Bar plot showing the percentage of drifting-grating responding neurons across visual regions, with neurons demonstrating exclusive positive responses represented in orange, exclusive negative responses in teal, and neurons with non-exclusive responses in gray.(B) Bar plot displaying the count of positive-delay neurons and negative-delay neurons within the population of enhanced neurons (left) and suppressed neurons (right). (C) The effects of pre-stimulus state (running / stationary) on the averaged stimulus-evoked response of enhanced neurons (top) and suppressed neurons (bottom) in the representative mouse. (D) Change in drifting-grating response between running and stationary. Single neuron response is quantified as the onset spiking rate (0 to 400ms) subtracted by the baseline (-800 to 0ms). Data is separated into two groups with orange color marked response of enhanced neurons and teal color marked that of suppressed neurons. Each dot within each group represents a mouse.
